# Selection of reference genes for qRT‐PCR and expression analysis of high‐altitude‐related genes in grassland caterpillars (Lepidoptera: Erebidae: *Gynaephora*) along an altitude gradient

**DOI:** 10.1002/ece3.3431

**Published:** 2017-09-25

**Authors:** Li Zhang, Qi‐Lin Zhang, Xiao‐Tong Wang, Xing‐Zhuo Yang, Xiao‐Peng Li, Ming‐Long Yuan

**Affiliations:** ^1^ State Key Laboratory of Grassland Agro‐Ecosystems College of Pastoral Agricultural Science and Technology Lanzhou University Lanzhou China; ^2^ State Key Laboratory of Pharmaceutical Biotechnology School of Life Sciences Nanjing University Nanjing China

**Keywords:** adaptive evolution, gene expression, natural population, reference gene, stability evaluation, Tibetan Plateau

## Abstract

Changes in gene expression patterns can reflect the adaptation of organisms to divergent environments. Quantitative real‐time PCR (qRT‐PCR) is an important tool for ecological adaptation studies at the gene expression level. The quality of the results of qRT‐PCR analysis largely depends on the availability of reliable reference genes (RGs). To date, reliable RGs have not been determined for adaptive evolution studies in insects using a standard approach. Here, we evaluated the reliability of 17 candidate RGs for five *Gynaephora* populations inhabiting various altitudes of the Tibetan Plateau (TP) using four independent (geNorm, NormFinder, BestKeeper, and the deltaCt method) and one comprehensive (RefFinder) algorithms. Our results showed that *EF1‐*α, *RPS15*, and *RPS13* were the top three most suitable RGs, and a combination of these three RGs was the most optimal for normalization. Conversely, *RPS2*,*ACT*, and *RPL27* were the most unstable RGs. The expression profiles of two target genes (*HSP70* and *HSP90*) were used to confirm the reliability of the chosen RGs. Additionally, the expression patterns of four other genes (*GPI*,*HIF1A*,*HSP20*, and *USP*) associated with adaptation to extreme environments were assessed to explore the adaptive mechanisms of TP *Gynaephora* species to divergent environments. Each of these six target genes showed discrepant expression patterns among the five populations, suggesting that the observed expression differences may be associated with the local adaptation of *Gynaephora* to divergent altitudinal environments. This study is a useful resource for studying the adaptive evolution of TP *Gynaephora* to divergent environments using qRT‐PCR, and it also acts as a guide for selecting suitable RGs for ecological and evolutionary studies in insects.

## 1 INTRODUCTION

Evolutionary shifts in gene expression profiles can be used to explore genetic targets involved in local adaptation and ecological speciation (Huang et al., 2016). Variation in gene expression is considered one of the most important mechanisms for the adaptation of animals to ecological environments (Eyres et al., 2016; Qu et al., 2013). Although next‐generation sequencing technologies are widely used in ecological and evolutionary studies (Eyres et al., 2016; Luo, Yang, & Gao, 2013), the data obtained from high‐through sequencing may still need to be further confirmed by quantitative real‐time PCR (qRT‐PCR) experiments. With its large dynamic range, high sensitivity, and reproducibility, qRT‐PCR is an effective technology for gene expression analyses, especially in species for which genomic information is lacking (Bansal et al., 2016; Sun et al., 2016). When using qRT‐PCR to compare gene expression patterns, the accuracy and reliability of the qRT‐PCR results are influenced by many factors, such as RNA extraction, reverse transcription, cDNA concentration, and PCR efficiency (Guénin et al., 2009). Therefore, qRT‐PCR data must be normalized using reference genes (RGs) to avoid nonspecific variation or errors (Guénin et al., 2009). Ideal RGs should be stably expressed under various experimental conditions (Chapman & Waldenström, 2014). Several traditional RGs, such as elongation factor 1 alpha (*EF1‐*α), β‐actin (β*‐ACT*), 18S ribosomal RNA (*18S*), and glyceraldehyde‐3‐phosphate dehydrogenase (*GAPDH*), have been widely used in qRT‐PCR studies (Koramutla, Aminedi, & Bhattacharya, 2016; Nakamura et al., 2016; Robledo et al., 2014). However, previous studies have shown that these RGs are not always stably expression under various experimental conditions (Chapman & Waldenström, 2014; Thellin, Elmoualij, Heinen, & Zorzi, 2009). Moreover, several studies have highlighted the importance of the optimal number of RGs (normalization factors, NFs) being experimentally determined when normalizing target genes (Bansal et al., 2016; Yang, et al., 2015; Zhang et al., 2016).

Recently, the selection of suitable RGs for qRT‐PCR has been widely undertaken in many insect groups, such as the Hemiptera (Bansal et al., 2016; Koramutla et al., 2016; Li et al., 2013; Maroniche et al., 2011), Diptera (Nakamura et al., 2016; Ponton et al., 2011), Coleoptera (Lord, Hartzer, Toutges, & Oppert, 2010; Rajarapu, Mamidala, & Mittapalli, 2012), Orthoptera (Van Hiel et al., 2009), and Lepidoptera (Fu et al., 2013; Tang, Zhang, Xue, & Yuan, 2016; Zhu et al., 2014). These studies were primarily conducted to select RGs that are stably expressed throughout different developmental stages, biological tissues, treatments, and environmental stresses (e.g., microbial infection and chemical stimulus) (Lord et al., 2010; Nakamura et al., 2016; Schaeck et al., 2016). They intensively focused on changes in gene expression associated with insecticide resistance, immunity, growth, and differentiation in laboratory populations. Conversely, the selection of suitable RGs in field populations is very limited, which impedes our understanding of ecological adaptation (e.g., high‐altitude adaptation) at the gene expression level. Compared to laboratory populations, organisms in the wild generally suffer from more diverse and complex ecological stressors, which may result in discrepancies with the best RGs selected in the laboratory. Therefore, it is necessary to evaluate the stability of candidate RGs in field populations under divergent environmental conditions.

**Figure 1:**
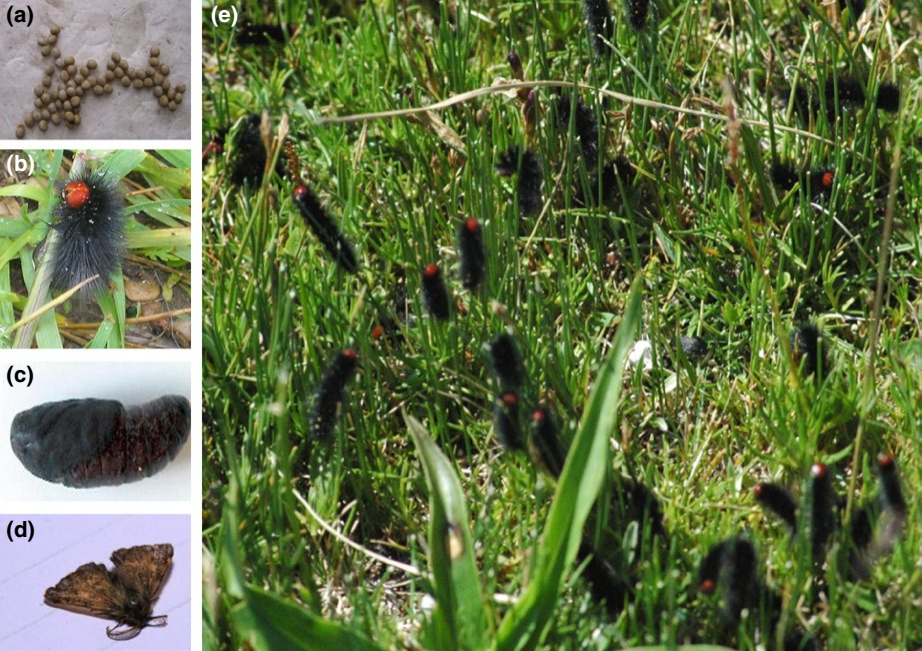
Images of four developmental stages of *Gynaephora*: (a) egg; (b) larvae; (c) pupa; (d) adult. Image of an Alpine meadow of the Tibetan Plateau with abundant *Gynaephora* larvae (e)

Grassland caterpillars (Lepidoptera: Erebidae: Lymantriinae: *Gynaephora*) are the most damaging insect pests in the alpine meadow of the Tibetan Plateau (TP) (Figure 1). Over the past 50 years, TP *Gynaephora* have caused enormous economic losses of forages (Zhang & Yuan, 2013). In particular, *Gynaephora* cocoons in meadows can cause mouth sores and broken tongue disease in domestic animals and wildlife (Yan, Wang, & Liu, 2006). To date, a total of 15 species are described for this small genus, of which eight are endemic to the TP, whereas the other seven species are mainly distributed in the high mountains and arctic areas of the northern hemisphere (Zhang & Yuan, 2013). *Gynaephora* first colonized the TP around 17.7 million years ago, when Tibet was located at an altitude 3,000 m above sea level (masl). A burst of speciation has been dated to 4.5–1.1 million years ago, which broadly corresponding with the rapid uplift of the TP (Yuan et al., 2015). Changes in altitude and related environmental factors were likely a driving force for the diversification of the *Gynaephora*. Therefore, TP *Gynaephora* species are ideal for investigating gene expression changes induced by divergent local environmental conditions. The harsh environment of the TP is typically characterized by hypoxia, low temperature, and high ultraviolet (UV) radiation, which imposes strong selective pressures on TP species (Luo et al., 2013; Yu et al., 2016). The molecular mechanisms underlying high‐altitude adaptation have been extensively studied in TP animals, such as humans (Huerta‐Sánchez et al., 2014; Yi et al., 2010), Tibetan mastiffs (Gou et al., 2014; Li et al., 2014), yaks (Qiu et al., 2012), Tibetan antelopes (Ge et al., 2013), snub‐nosed monkeys (Yu et al., 2016; Zhou et al., 2016), ground tits (Qu et al., 2013), and Tibetan cashmere goat (Song et al., 2016). However, genetic information regarding the gene expression changes that occur with adaptation to divergent high‐altitude environments in insects is largely lacking.

Here, we determined the suitability of 17 candidate RGs (Table 1) and recommended the optimal NFs for gene expression studies in five *Gynaephora* populations along an altitudinal gradient. The stability of the expression of these genes was evaluated using four independent statistical algorithms (geNorm, NormFinder, BestKeeper, and the deltaCt method) and one comparative method (RefFinder). Two target genes, encoding the heat shock 70 kDa protein (*HSP70*) and the heat shock 90 kDa protein (*HSP90*), were chosen to confirm the reliability of the selected RGs using three normalization strategies (recommended, optimal, and worst NFs). In addition to *HSP70* and *HSP90*, we also performed qRT‐PCR analyses of four other target genes encoding glucose‐6‐phosphate isomerase (GPI), hypoxia‐inducible factor 1 alpha (HIF1A), heat shock 20 kDa protein (HSP20), and ubiquitin‐specific protease (USP) using the optimal RGs (*EF1‐*α, *RPS15*, and *RPS13*) for normalization. Previous studies showed that these six target genes were associated with the response to divergent altitude stressors in TP animals, such as *Rana kukunoris* (Yang, Qi, Bi, & Fu, 2012) and *Capra hircus* (Tang et al., 2015). This study provided an important resource for gene expression analyses of target genes in field populations of TP *Gynaephora* species, which will be helpful for further understanding the mechanisms of gene expression‐mediated adaptation in TP insects.

**Table 1:** Description of 17 candidate reference genes and six target genes in *Gynaephora* used for qRT‐PCR analysis

Gene name	Abbreviation	Primer name	Sequences (5′–3′)	Amplicon size (bp)	Tm	*E* (%)/*R* ^2^
18S ribosomal RNA	*18S*	18SF	ATTACCACAGTTATCCAA	138	54	98.7/.984
		18SR	CAGTAGTTATATGCTTGTC			
28S ribosomal RNA	*28S*	28SF	CTACTCGTTACGGCTTAATG	80	59	98.3/.994
		28SR	TGAAGCGTTTTGCCTATAC			
Actin	*ACT*	ActinF	AGAGGGAAATCGTGCGTGAC	195	60	100.3/.989
		ActinR	CCATACCCAAGAAGGAAGGC			
Arginine kinase	*AK*	AKF	TATGACATCTCCAACAAG	93	54	78.1.3/.993
		AKR	TTCAATCTTAATCAGTTCAG			
Cyclin A	*CYCA*	CyclinAF	GAATCTCCTATGTCAGTTGTG	94	59	111.1/.983
		CyclinAR	CAGTGGTTGTGTCTTCATC			
Elongation factor 1 alpha	*EF‐1*α	EF1αF	CCCGCCAACATCACCACT	130	60	97.3/.972
		EF1αR	CGTAACCACGACGCAACTCC			
Glyceraldehyde‐3‐phosphate dehydrogenase	*GAPDH*	GAPDHF	GTGGAATCTACTGGTGTAT	85	57	120.6/.997
		GAPDHR	GAGCAGAGATGATGACTT			
Ribosomal protein L10	*RPL10*	RPL10F	CATTCTAATGTGGAACTGAT	114	57	96.7/.990
		RPL10R	CTTGTGTCTGACGAGTAT			
Ribosomal protein L27	*RPL27*	RPL27F	CACTCTTGTATCTTTCCT	116	54	111.1/.993
		RPL27R	TTACTCAGTAGACTTCAG			
Ribosomal protein L28	*RPL28*	RPL28F	CCTTAGCCTTCTTGTATA	110	53	119.7/.989
		RPL28R	GACTAACCTCAACTCTTA			
Ribosomal protein S2	*RPS2*	RPS2F	CTTGGCAAGTATGATAGC	84	56	114.5/.994
		RPS2R	GACAACAATGGACACATC			
Ribosomal protein S13	*RPS13*	RPS13F	TGACTTGTGCTACTCCAT	110	59	105.2/.984
		RPS13R	ACTGACTGCTGATGATGT			
Ribosomal protein S15	*RPS15*	RPS15F	GTTGGCTCTATTGTAGGTATC	108	59	99.7/.984
		RPS15R	AGGCTTGTATGTGACTGA			
Troponin C	*TPNC*	TPNCF	TGCCAAGTTCATCGTAGA	94	59	95.8/.990
		TPNCR	AATGTAACCGTTGCCTTC			
α‐Tubulin	α*‐TUB*	α‐TubulinF	GACCTCATCAACTACTGT	95	57	111.3/.999
		α‐TubulinR	ACTCCTTCAACACATTCT			
β‐Actin	β*‐ACT*	β‐actinF	TATGGAATCTTGCGGTATC	76	58	106.7/.976
		β‐actinR	CAAGTCCTTACGGATGTC			
β‐Tubulin	β*‐TUB*	β‐TubulinF	CTGCGATATTCCTCCTAA	95	56	144.7/.984
		β‐TubulinR	ATTGCTCTGATATTCTCTTG			
Heat shock protein 70^a^	*HSP70*	HSP70F	CCAACAACACAGTCTTCG	75	60	151.5/.997
		HSP70R	TCATGTCCTGCTGAATCTTA			
Heat shock protein 90^a^	*HSP90*	HSP90F	ACAATACAGCAAGGTGAT	118	57	156.9/.997
		HSP90R	GTGAGGAAGACAAGGTTA			
Glucose‐6‐phosphate isomerase^a^	*GPI*	GPIF1	CTACTCGCTAACTTCTTG	86	56	95.6/.982
		GPIR1	CCTGACTTCTCTAACTCT			
Hypoxia‐inducible factor 1 alpha^a^	*HIF1A*	GynHIF1F	GACTTGTTGCTTCGTATGA	92	59	119.9/.993
		GynHIF1R	CCTGTGATGTGTATTACCTTAT			
Heat shock protein 20^a^	*HSP20*	HSP20F	TGAAGTGATTCGCCAAGA	135	60	87.1/.982
		HSP20R	AATTCCAAGTGAACCTAAGC			
Ubiquitin‐specific protease^a^	*USP*	USPF	TCGTAGAACAGTAGGTAGG	80	59	107.4/.997
		USPR	TGGTACACATCAGACACA			

*E*, PCR efficiency; *R* ^2^, correlation coefficient. Tm, annealing temperature

a Six target genes.

## 2 MATERIALS AND METHODS

### 2.1 Sampling

*Gynaephora* larvae (4th instar) were collected from the alpine meadow of the TP across five sampling sites (Huangchen: HC, Zeku: ZK, Yushu: YS, Naqu: NQ, and Anduo: AD) with different altitudes (3,100–4,800 masl). Detailed sampling information is provided in Table S1. Instar larvae were identified by the method of Yan et al.(2006). All samples were collected in the field, immediately frozen in liquid nitrogen, and stored at −80°C at the State Key Laboratory of Grassland Agro‐Ecosystems, Lanzhou University, Lanzhou, China.

### 2.2 RNA extraction and cDNA synthesis

For each locality, total RNA was isolated from a pool of five 4th‐instar larvae using TRIzol reagent (Ambion, USA) according to the manufacturer's instructions. Residual genomic DNA was digested by RNase‐free DNase (Qiagen, Germany) according to the manufacturer's instructions. Three biological replicates were performed for each locality. RNA quality and quantity were measured with a Nanodrop 1000 spectrophotometer (Thermo Scientific, USA), and its integrity was confirmed by 1.5% agarose gel electrophoresis. Only high‐quality RNA with an OD_260_/OD_280_ between 1.8 and 2.0 was used for subsequent analyses. Single‐stranded cDNA was synthesized in a total volume of 20 μl containing 4 μl 5× PrimeScript RT Master Mix (including oligo dT primers; TaKaRa, Japan), 1 μl total RNA (1 μg/μl), and 15 μl RNase‐free dH_2_O under conditions of 37°C for 15 min and 85°C for 5 s, according to the manufacturer's protocol. cDNA (100 ng/μl) diluted with Rnase‐free water was used for further experiments.

### 2.3 Design and evaluation of primers for each gene

Seventeen candidate RGs were selected for assessment of robustness as internal controls for qRT‐PCR. Six target genes (*HSP70*,* HSP90*,* GPI*,* HIF1A*,* HSP20*, and *USP*) were analyzed to explore changes in gene expression in the five TP *Gynaephora* populations from divergent altitudinal environments (Table 1). All sequences of reference and target genes were obtained from the annotated transcriptome of *G. menyuanensis* (Table S2). All specific primers were designed using Beacon Designer 7.0. The specificity of the amplification product for each primer was checked by the appearance of a single band at the targeted length using 1.5% agarose gel electrophoresis. We also confirmed all gene‐specific amplified PCR products by sequencing. The amplification efficiency and correlation coefficient (*R* ^2^) of each primer were calculated using the standard curve generated from a 10‐fold dilution series of mixed cDNA samples at five dilution. The corresponding qRT‐PCR efficiencies (*E*) were calculated according to the equation *E* (%) = (10^(−1/slope)^ − 1) × 100 (Zhang et al., 2016).

### 2.4 Quantitative RT‐PCR

qRT‐PCR was performed using an ABI7500 real‐time PCR system (Applied Biosystems, USA). cDNA was amplified using the SYBR Premix Ex Taq II (TaKaRa, Japan) according to the manufacturer's protocol. Reactions were prepared in a total volume of 10 μl containing 1 μl diluted cDNA (100 ng/μl), 5 μl 2 × SYBR Premix ExTaq II (TaKaRa, Japan), 3 μl RNase‐free sterile water, and 0.5 μl each of the forward and reverse primers (10 ng/μl). The PCR program was 95°C for 30 s, 40 cycles of 95°C for 5 s, 55°C for 30 s, and 72°C for 30 s, followed by a melting curve analysis to confirm the specificity of amplification for each reaction. The reaction solution without a cDNA template was used as a negative control to confirm template‐specific amplification. PCR reactions were conducted for three biological replicates, and the detection of each gene was performed in an independent sample with three technical replicates.

### 2.5 Determining the stability of candidate RGs expression

The expression stability of each RG was evaluated with four independent algorithms: geNorm (Vandesompele et al., 2002), NormFinder (Andersen, Jensen, & Ørntoft, 2004), BestKeeper (Pfaffl, Tichopad, Prgomet, & Neuvians, 2004), and the deltaCt method (Silver, Best, Jiang, & Thein, 2006). All data from three biological and technological replicates were used to calculate the average Ct value according to previous methods (Yang et al., 2015; Zhang et al., 2016). geNorm software calculates an expression stability value (*M*) and ranks the genes in order for a given set of samples. A lower *M* value indicates a higher expression stability. Pairwise variation (*V*) analysis was performed to evaluate the most reliable NFs (Vandesompele et al., 2002). The pairwise variation value of *V*_*n*_/*V*_*n*+1_ between two sequential NFs was used to determine the optimal number of RGs required for better normalization. A threshold value below 0.15 suggests that no additional RGs are needed for normalization (Vandesompele et al., 2002). NormFinder ranks candidate RGs by calculating their stability values, with lower values indicating more stable genes (Andersen et al., 2004). NormFinder calculates not only the overall variation of the candidate normalization genes but also the variation between sample subgroups of the sample set (Andersen et al., 2004). The BestKeeper program determines the stability of an RG based on the standard deviation (*SD*) of the Ct values, with a lower *SD* (Pfaffl et al., 2004) indicating a more stable RG. The deltaCt method calculates the relative expression levels (RELs) between one RG and the other RGs within each sample, with candidate RGs with smaller *SD* values of RELs being more stable (Silver et al., 2006). Finally, the candidate genes were ranked based on the web‐based analysis tool RefFinder (http://www.leonxie.com/referencegene.php) (Xie, Sun, Stiller, & Zhang, 2011). RefFinder ranks all RG candidates based on the main statistics methods used, including those from geNorm, NormFinder, BestKeeper, and the deltaCt method (Silver et al., 2006), and then finally ranks the RGs by calculating the geometric mean (GM) values. RGs with lower GM values are considered to be more stable. Thus, RefFinder enables the best candidates to be selected based on the comprehensive ranking results of different programs.

### 2.6 Evaluation of suitable RGs

To confirm the reliability of the RGs, the RELs of *HSP70* and *HSP90* were determined and normalized using the most stable RG [*EF‐1*α = NF(1)], the three top stable RGs [*EF‐1*α, *RPS15*, and *RPS13 *=* *NF(1–3)], and the two least stable RGs [*ACT*,* RPL17 *=* *NF(16–17)]. Relative normalized expression values were calculated for each gene using the 2^−ΔΔCt^ method (Livak & Schmittgen, 2001; Pfaffl et al., 2004). Statistical analysis of the data was performed using the IBM SPSS statistics 22 software based on the nonparametric Mann–Whitney *U* test.

### 2.7 Quantitative RT‐PCR analysis of six target genes associated with high‐altitude adaptation

The RELs of six target genes were explored based upon the optimal RGs [*EF‐1*α, *RPS15*, and *RPS13 *=* *NF(1–3)]. The method of calculation and statistical analysis of the relative quantification of these target genes were the same as described above. The data obtained from biological replicates were analyzed separately to verify that the variation was not due to the treatment but was intrinsic to the gene itself (Castro, Román, Rubio, & Die, 2012; Remans et al., 2008).

## 3 RESULTS

### 3.1 Efficiency of primers for candidate RGs

Quantitative real‐time PCR amplification with each primer pair yielded a single specific band of the expected size following 1.5% agarose electrophoresis (Fig. S1). The melting curve of the qRT‐PCR showed a single peak for each primer pair, indicating the absence of any nonspecific amplification (Fig. S2). The amplification efficiency (*E*%) values of all candidate RGs ranged from 78.1 to 144.7, with correlation coefficient (*R* ^2^) values varying from .972 to .999 (Table 1).

### 3.2 Determination of expression stability of candidate RGs

**Figure 2:**
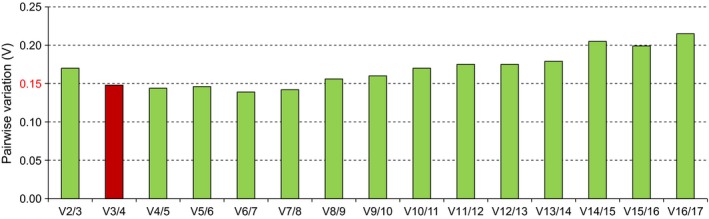
Pairwise variation (*V*_*n*_/*V*_*n*+1_) analysis for selecting the optimal number of reference genes in the normalization of five *Gynaephora* populations using the geNorm algorithm. Values <0.15 indicate that additional genes are not required for gene expression analysis, and the optimal number of normalization factors is indicated in red

The geNorm algorithm indicated that *RPL10* and *RPS13* were the most stable genes, followed by *EF‐1*α, β*‐ACT*,* TPNC*, and *RPS15* (Table 2).The optimal number of suitable RGs required for proper normalization was determined to be fewer than three based upon a *V*_3/4_ value of 0.148 (with <0.15 being the default cutoff) (Figure 2), suggesting that three RGs could be used for normalization. NormFinder analysis identified *EF‐1*α as the most stable, followed by *RPS15* and *TPNC*, whereas *RPL27* was the least stable RG (Table 2). According to BestKeeper analysis, *28S* was the most stable gene, followed by *CYCA* and *18S*, while *ACT* was the least stable (Table 2). The best RG according to the deltaCt method as *EF‐1*α, whereas *RPL27* and *18S* showed the lowest expression stabilities, similar to the results of the NormFinder analysis (Table 2). The results of a comprehensive ranking using RefFinder showed that the most stable RGs were *EF‐1*α, *RPS15*, and *RPS13*, whereas the least stable were *AK*,* 18S*,* RPS2*,* ACT*, and *RPL27* (GM > 9.0) (Table 2).

**Table 2:** Stability ranking of the candidate reference genes in the five *Gynaephora* populations derived from different altitudes

Reference gene	RefFinder	geNorm		NormFinder		BestKeeper		deltaCt	
	Rank	SV	Rank	SV	Rank	*SD*	Rank	SV	Rank
*EF‐1*α	1	0.417	2	0.52	1	3.01	8	1.68	1
*RPS15*	2	0.725	5	0.58	2	2.64	6	1.80	3
*RPS13*	3	0.214	1	1.06	6	3.22	11	1.83	4
*TPNC*	4	0.625	4	0.63	3	3.04	9	1.75	2
*RPL10*	5	0.214	1	1.10	8	3.19	10	1.85	6
β*‐ACT*	6	0.529	3	1.07	7	3.43	13	1.84	5
β*‐TUB*	7	1.05	8	0.91	4	2.84	7	1.99	8
*RPL28*	8	1.186	9	0.97	5	2.43	5	2.06	9
*28S*	9	1.986	14	2.80	15	0.51	1	3.22	15
*CYCA*	10	1.789	13	2.06	12	1.87	2	2.67	12
α*‐TUB*	11	1.486	11	1.72	11	2.16	4	2.48	11
*GAPDH*	12	0.818	6	1.14	9	3.53	14	1.93	7
*AK*	13	0.911	7	1.50	10	3.67	15	2.14	10
*18S*	14	2.164	15	2.96	16	1.95	3	3.33	16
*RPS2*	15	1.639	12	2.62	14	3.36	12	3.00	14
*ACT*	16	1.327	10	2.50	13	4.39	17	2.80	13
*RPL27*	17	2.371	16	3.62	17	3.88	16	3.93	17

SV, stability value.

### 3.3 Expression analysis of target genes for RG validation

**Figure 3:**
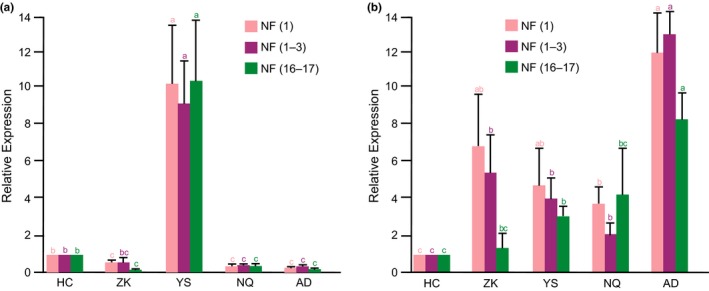
Expression profiles of *HSP70* (a) and *HSP90* (b) based on different normalization factors in five *Gynaephora* populations derived from different altitudes. Normalization was performed using the most stable RGs [*EF‐1*α; NF(1)], the top three most stable RGs [*EF‐1*α, *RPS15*,*RPS13*; NF(1–3)], and the two least stable RGs [*ACT*,*RPL27*; NF(16–17)]. Data are shown as mean ± standard deviation of three biological replicates. Significant expression differences among the five populations are indicated by different lowercase letters in the same color (*p *<* *.05), as determined by the nonparametric Mann–Whitney *U* test. RGs, reference genes; NF, normalization factor

Overall, the expression levels of *HSP70* and *HSP90* peaked in the YS and AD populations, respectively (Figure 3). *HSP70* had the lowest expression in the AD population when using NF(1) or NF(1–3) for normalization, but it had the lowest expression level in the ZK population using NF(16–17). Compared to expression levels in the HC population, the expression of *HSP70* was significantly down‐regulated in the ZK population based on NF(1) or NF(16–17) normalization but not NF(1–3). The expression of *HSP90* was significantly up‐regulated in the ZK and NQ populations based on NF(1) and NF(1–3) normalization but not using NF(16–17). Although there were no significant differences among the ZK, YS, and NQ populations, the expression of *HSP90* gradually decreased along with an increasing altitudinal gradient when using NF(1) and NF(1–3) for normalization, whereas a gradual increase in expression was observed using NF(16–17). The difference between the highest expression level of *HSP70* (YS) and the lowest (ZK) was 103‐fold when using NF(16–17) for normalization. However, using NF(1–3) or NF(1) for normalization of *HSP70*, the difference between the highest expression level (YS) and the lowest (AD) was 52‐ or 50‐fold, respectively (Figure 3)**.** Based on these findings, the normalization results based on NF(16–17) did not accurately reflect the expression levels of the two target genes in the five *Gynaephora* populations distributed among divergent altitudinal environments, indicating the necessity of selecting stable RGs and using multiple internal genes for normalization of qRT‐PCR experiments.

### 3.4 Expression analysis of high‐altitude‐related genes requires normalization with the optimal RGs

**Figure 4:**
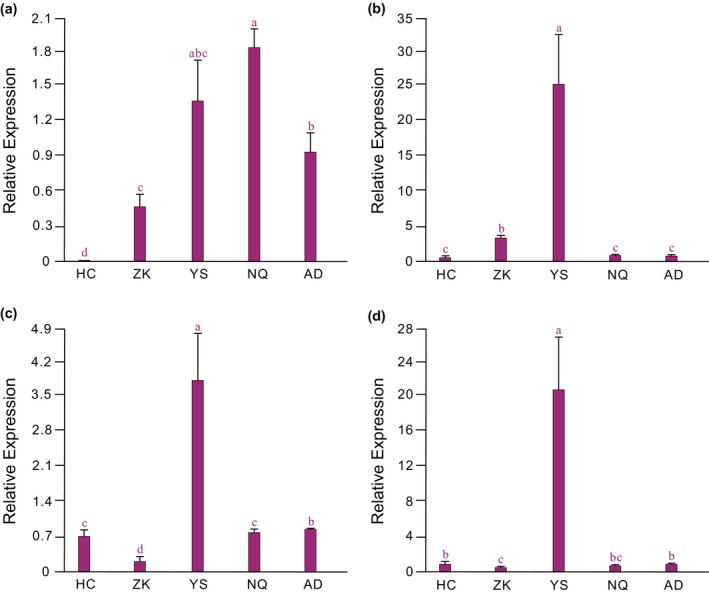
Gene expression analysis of four genes associated with high‐altitude adaptation. Normalization was performed using the top three most stable RGs [*EF‐1*α, *RPS15*,*RPS13*; NF(1–3)]. (a) Glucose‐6‐phosphate isomerase (*GPI*); (b) hypoxia‐inducible factor 1 alpha (*HIF1A*); (c) heat shock protein 20 (*HSP20*); (d) ubiquitin‐specific protease (*USP*). Bars indicate the standard deviation (±*SD*) of three biological replicates. Significant expression differences among the five populations are indicated by different lowercase letters (*p *<* *.05), as determined by the nonparametric Mann–Whitney *U* test. RGs, reference genes; NF, normalization factor

A significant difference in the expression level of each of the six target genes (*GPI*,* HIF1A*,* HSP20*,* HSP70*,* HSP90*, and *USP*) was found between the HC population and most other populations (Figures 3 and 4). These genes showed expression patterns that varied depending on altitude. With exception of *GPI* in the NQ population and *HSP90* in the AD population, the highest expression for each of the other four genes was found in the YS population. Compared to the HC population, the YS population showed significantly up‐regulated expression of all six genes. Three genes (*HSP90*,* GPI*, and *HSP20*) were significantly up‐regulated, and *HSP70* was significantly down‐regulated in the AD population, whereas the expressions of the remaining two genes (*HIF1A* and *USP*) did not differ significantly between the HC and AD populations. Expressions of *GPI* and *HIF1A* increased from the HC population to the NQ and YS populations and then declined in the AD and NQ populations, respectively. *HSP90* showed a relatively smaller peak in expression in the ZK population and then continued to decrease in the YS and NQ populations. Interestingly, *HSP20* and *USP* were significantly down‐regulated in the ZK population and then significantly up‐regulated in the YS population, and finally significantly down‐regulated in the NQ and AD populations.

## 4 DISCUSSION

As suggested in the “Minimum Information for publication of Quantitative real‐time PCR Experiments” (MIQE) guideline (Bustin et al., 2009), accurate results strongly depend on the availability of reliable RGs. Expression of the optimal RGs should be stable under various conditions, such as in different tissues, treatment conditions, and cell lines (Robledo et al., 2014). However, due to the noncommonality of optimal RG and NF numbers in qRT‐PCR experiments, these should be determined under specific experimental conditions (Bustin et al., 2009; Vandesompele et al., 2002). Fortunately, several statistical algorithms now provide the ability to assess the expression stability of candidate RGs. In this study, comparative analysis showed high consistency in the ranking of 17 candidate RGs according to stability among different statistical methods. For example, the results from the deltaCt method were highly similar to those of NormFinder. However, due to different statistical models among algorithms, some discrepancies were also observed among the four independent algorithms, as has been frequently reported in previous studies (Ibanez & Tamborindeguy, 2016; Koramutla et al., 2016; Yang, et al., 2015). The BestKeeper results were the most divergent from the other three independent algorithms, as reported in other studies (Lu et al., 2015; Zhang et al., 2016). In order to overcome differences among algorithms, we determined an overall ranking for the 17 candidates RGs based on another algorithm (RefFinder) to obtain the final stabilities. Our results suggest that additional research involving more candidate RGs under various experimental conditions with more statistical algorithms is needed to improve the reliability and accuracy of qRT‐PCR.

*EF‐1*α was the best RG for grassland caterpillars from divergent altitudinal environments, as observed in other lepidopteran insects, such as *Spodoptera litura* under different developmental stages (Zhu et al., 2014), *Plutella xylostell* under diverse experimental conditions (developmental stages, tissues, and strains) (Fu et al., 2013), larval *Sesamia inferens* under insecticide exposure (Lu et al., 2015), and *Plodia interpunctella* under different strains (Tang et al., 2016). This reflects the stable expression of *EF‐1*α in lepidopteran insects under many experimental conditions, and this gene should be considered as a candidate RG in the gene expression analysis of lepidopteran insects in the future. In addition to lepidopterans, *EF‐1*α has also performed well as an RG in other insects, such as *Anastrepha oblique* under different developmental stages (Nakamura et al., 2016), *Bemisia tabaci* across various abiotic conditions (Li et al., 2013), *Drosophila melanogaster* under heat‐stressed conditions (Ponton et al., 2011), *Dicentrarchus labrax* under nutritional/environmental changes (Schaeck et al., 2016), and *Bactericera cockerelli* under different life stages (Ibanez & Tamborindeguy, 2016). Nevertheless, *EF‐1*α has also exhibited unstable expression in some insects, such as rice planthopper (*Delphacodes kuscheli*) under viral infection conditions (Maroniche et al., 2011).

Two ribosomal protein genes (*RPS15* and *RPS13*) also showed stable expressions among five *Gynaephora* populations, as observed in *Helicoverpa armigera* under different developmental stages and tissues (Zhang et al., 2014). *RPS15* had been demonstrated to be a RG in humans (Kitagawa et al., 1991; Shiga, Yamamoto, & Okamoto, 1990) and is a suitable RG in many cases in mammals (Bionaz & Loor, 2007; Kumar et al., 2012). Interestingly, the least stable genes according to our stability ranking included some genes widely used as RGs in previous studies (e.g., *ACT* and *18S*). Although *TPNC* has not always been considered as a stable RG in general, in the present study, this gene ranked among the top four in terms of stability. Therefore, unconventional RGs (e.g., *TPNC*) should not be ignored as candidates in further experiments involving RG selection. These results indicated that the selection of properly tested RGs is necessary prior to conducting experiments.

As suggested by several studies, the accuracy of qRT‐PCR can be improved by using more than one RG (Vandesompele et al., 2002; Yüzbaşıoğlu, Onbaşılar, Kocaefe, & Özgüç, 2010). The optimal number of NFs for normalization was evaluated by pairwise variation analysis using geNorm. The validation of two target genes (*HSP70* and *HSP90*) also indicated that the combination of three RGs (NF = 3, *EF‐1*α, *RPS15*, and *RPS13*) was sufficient for optimal normalization. Conversely, the least stable candidate genes (*ACT*,* RPL2*, and *RPL27*) were not suitable for the normalization of the qRT‐PCR data of grassland caterpillars from different altitudes, despite using the same number of NFs. Overall, it should be noted that the optimal number of RGs will vary under different experimental conditions, and therefore, these genes should be experimentally selected for each study according to the MIQE guideline.

Several studies have shown that adaptation to ecological stressors not only involves genomic structural and sequence variations (positive selection), but also variation in gene expression levels (Tang et al., 2015; Zhao et al., 2013). Here, we performed qRT‐PCR analyses normalized using the optimal RGs to detect the expression patterns of six target genes (*GPI*,* HIF1A*,* HSP20*,* HSP70*,* HSP90*, and *USP*) associated with adaptation to extreme environments. Previous studies have shown that variation in the expression of *GPI* can improve the ability of organisms to adapt to extreme environments, such as high salinity and hypoxia (Cui et al., 2010; Naughton, 2003). Low oxygen supply is a major challenge for species living at high altitudes (Yang, Wang, Zhang, & He, 2015); thus, hypoxia may be a key ecological factor driving changes in *GPI* expression in the grassland caterpillar. The expression responses of *GPI* to divergent altitudes might be key in the adaptation of the energy metabolism of TP *Gynaephora* species. *HIF1A* is a main regulator in the hypoxia signaling pathway, and responses to hypoxia are largely regulated by changes in its expression in other animals (Wang et al., 2015; Xiao, 2015). Here, we observed significant expression changes in *HIF1A* along an altitudinal gradient, suggesting that the expression of this gene may be associated with adaptation to hypoxia in *Gynaephora*. Whether the gene sequence of *HIF1A* has also experienced adaptive evolution in *Gynaephora* remains unknown, and further study of the population genetics of this gene involving DNA sequencing techniques is required. Notably, we investigated *HIF1A*‐related genes through automated computational analysis of the NCBI database and found *HIF1A* genes in Hemiptera, Hymenoptera, and Ephemeroptera (data not shown), indicating that HIF1A may be important in the adaptation of insects to divergent environments.

Low temperature is also a severe ecological stress for TP *Gynaephora* species. HSPs have been shown to be involved in cryoprotection in insects under cold conditions (Singh, Jaiswal, & Sharma, 2013). Three genes in the HSP family (*HSP20*,* HSP70*, and *HSP90*) showed significant differences in expression across the five *Gynaephora* populations. *HSP70* is the most commonly studied transcript expressed after cold shock in many insects (Singh et al., 2013). Although the specific functional mechanism of *HSP70* in insects has not been studied under low temperature, more intense cold shocks induce high expression of *HSP70* that is more significant and of longer duration, and its up‐regulation may increase survival rates following cold exposure in insect diapauses (Li, Andorfer, & Duman,^1998^; Singh et al., 2013). *HSP90* recognizes and repairs damaged proteins that are bound by the constitutive form of *HSP70*, sequestering heat shock transcription factor and then degrading the target protein (Cheng et al., 2016; Singh et al., 2013). *HSP90* is also up‐regulated in response to cold shock in insects (Wu et al., 2017). Small HSPs (sHSPs) are a highly diverse family of proteins (12–40 kDa) (Singh et al., 2013). sHSPs show high heterogeneity and are identified by a conserved alpha‐crystalline domain (Singh et al., 2013; Zhu et al., 2013). Previous studies of thermal tolerance in insects have reported the response of *HSP20* expression to changes in temperature (King & MacRae, 2015). Here, we found that *HSP20* expression was strongly affected by differences in altitudinal environments. Therefore, the HSP family may influence cold tolerance in TP *Gynaephora* species at various altitudes. Furthermore, the up‐regulated expression of HSPs has been detected in hypoxia‐induced and UV‐radiated insects, which is thought to be responsible for reducing reactive oxygen species (Azad, Ryu, & Haddad, 2011; King & MacRae, 2015). These studies indicate that ecological stressors represent a complex and diverse challenge for high‐altitude animals.

Ultraviolet radiation is one of the three most serious challenges for the survival of animals on the TP (Yang et al., 2012). *USP* is a conserved gene that encodes an ubiquitin‐specific protease that plays a key role in DNA repair against UV damage (Yang et al., 2012). Our results showed that *USP* expression was significantly up‐regulated in the YS population compared with levels at lower altitudes, indicating that the expression of this gene responded dramatically to the rising altitude. Thus, *USP* may be particularly important for the resistance of *Gynaephora* to the high UV radiation from the exposure to sunlight acquired by climbing on the top of the grass or forage leaves.

Previous studies have reported a general reduction in nuclear gene expression during the entry into hypometabolic states in stress‐tolerant animals based on an energy‐saving mechanism (McMullen & Storey, 2008; Storey & Storey, 2004); a similar suppression of mitochondrial gene transcription was detected in the *Epiblema* moth (McMullen & Storey, 2008). Therefore, we proposed that the transcriptional suppression of these target genes in responses to extreme environments may ensure a proper energy storage when faced with ecological challenges beyond a certain range. Our results showed that most target genes reached a peak of transcription in the YS population, expression suppression initially occurring between altitudes of 4,000 (YS) to 4,500 masl (NQ). Thus, in the future, comparisons of gene expression differences between the YS and NQ populations will be valuable for exploring the adaptation of TP insects to high‐altitude environments using RNA‐Seq of additional *Gynaephora* populations living at divergent altitudes. The use of the most reliable RGs and number of NFs for normalization should improve our understanding of the molecular mechanisms involved in insect adaptation to divergent altitudinal environments.

## 5 CONCLUSION

In this study, we selected and validated suitable RGs for ecological and evolutionary studies in natural populations of TP insects. As a case study, we assessed the expression levels of six well‐known genes involved in adaptation to extreme environments. Our results confirmed the necessity of the evaluation of RG stability and the choice of appropriate RGs for ensuring the accuracy of qRT‐PCR results. The recommended RGs are expected to be important resources for gene expression analyses of target genes in natural populations of TP *Gynaephora* and other insects. In addition, measuring the expression levels of target genes associated with ecological adaptation using the most reliable RGs for normalization should provide insight into the molecular mechanisms associated with insect adaptation to divergent altitudinal environments.

## AUTHOR CONTRIBUTIONS

M.L.Y. designed the study. Q.L.Z. and M.L.Y. collected the insect samples. L.Z., X.T.W., X.Z.Y., and X.P.L. performed the molecular experiment. L.Z., Q.L.Z, X.T.W., and X.Z.Y. analyzed the data. L.Z. and Q.L.Z. wrote the manuscript. M.L.Y. revised the manuscript. All authors read and approved the final manuscript.

## CONFLICT OF INTEREST

None declared
