# Supplementary figures and images for: Selection of reference genes for qRT‐PCR and expression analysis of high‐altitude‐related genes in grassland caterpillars (Lepidoptera: Erebidae: Gynaephora) along an altitude gradient
Source: Ecol Evol. 2017 Sep 25;7(21):9054–65. doi: 10.1002/ece3.3431 (PMC5677504; doi:10.1002/ece3.3431)

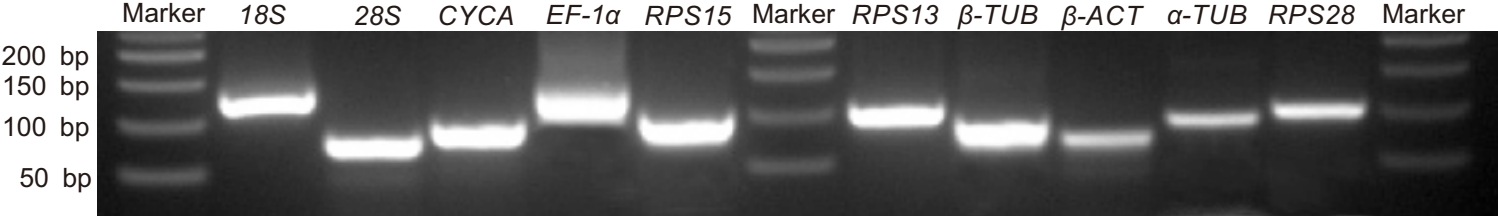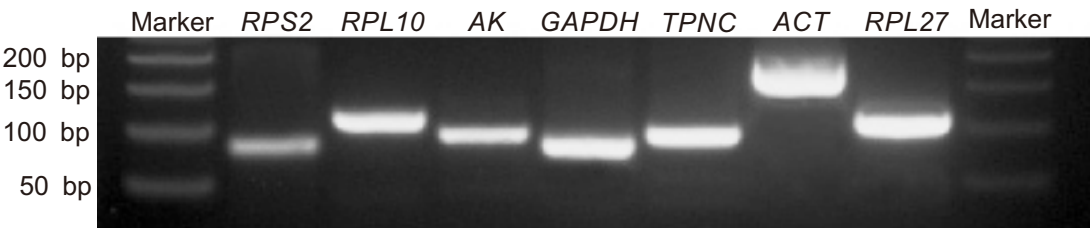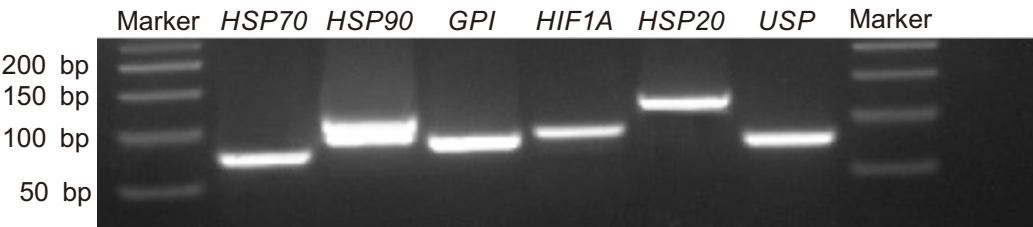

Supplement: Supplementary file 1 [file ECE3-7-9054-s001.pdf]

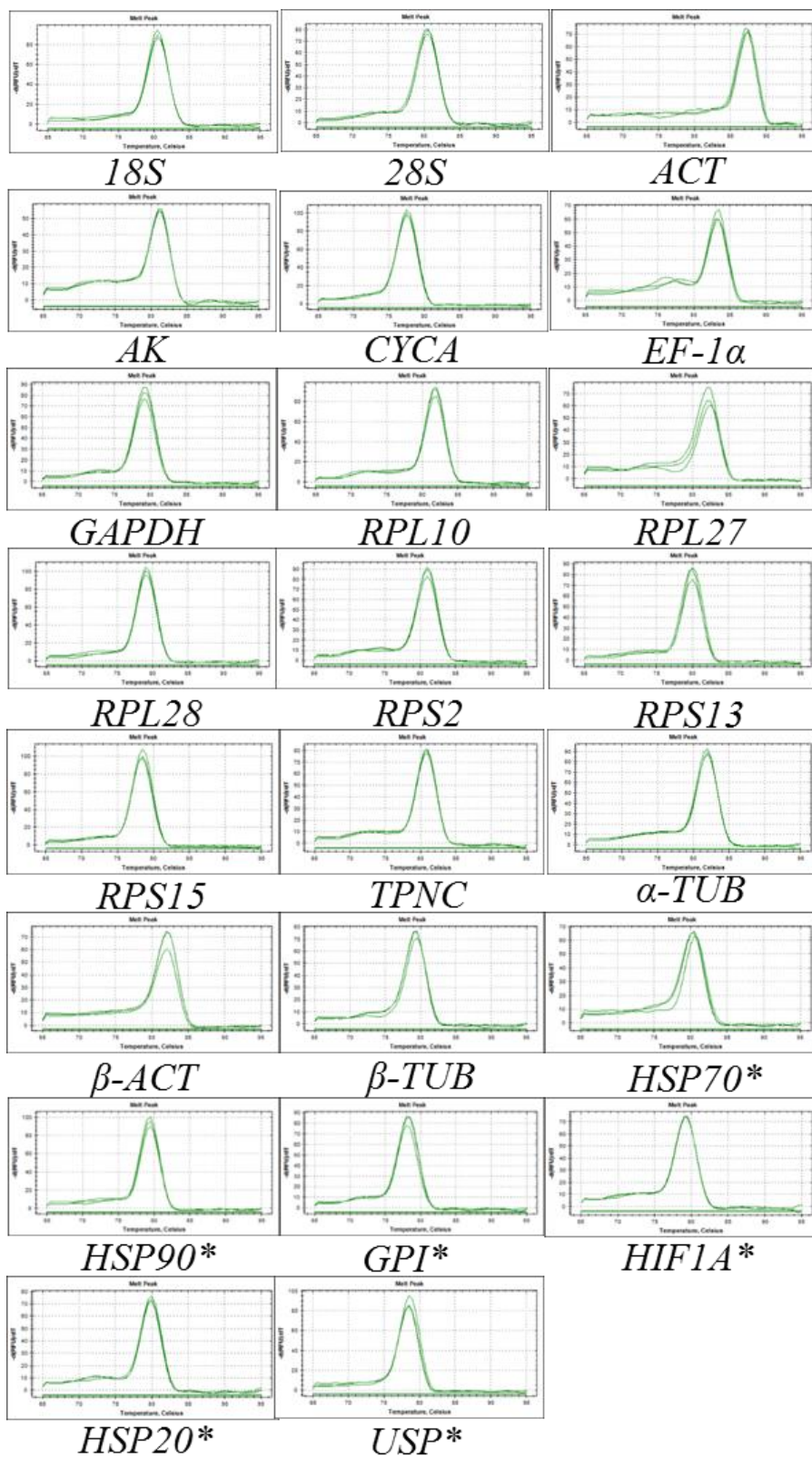

Supplement: Supplementary file 2 [file ECE3-7-9054-s002.pdf]
