# Supplementary material for: Selection of reference genes for qRT‐PCR and expression analysis of high‐altitude‐related genes in grassland caterpillars (Lepidoptera: Erebidae: Gynaephora) along an altitude gradient
Source: Ecol Evol. 2017 Sep 25;7(21):9054–65. doi: 10.1002/ece3.3431 (PMC5677504; doi:10.1002/ece3.3431)
Supplement: Supplementary file 4 [file ECE3-7-9054-s004.docx]

**Table S2** **Sequence information for PCR amplified candidate reference genes from the transcriptome of *Gynaephora* *menyuanensis***.

>*18S ribosomal RNA* (*18S*)

TAATGATCCTTCCGCAGGTTCCCCTACGGAAACCTTGTTACGACTTTTACTTCCTCTAAATGATCAAGTTTGGTCAACTTCCCAGCAACGCCGACGGCCGTGAAGCCACCGCGTGTCGGTCCGAAGACCTCACTAAATCATTCAATCGGTAGTAGCGACGGGCGGTGTGCACAAAGGGCAGGGACGTAATCAACGCGAGCTTATGACTCGCGCTTACTAGGAATTCCTCGTTTATGGGGGATAATTGCAAACCCCAATCCCCAGCACGAAGGAGTTTCAGCGGGTTGCCCGGGCCTCTAGGCCAGGGAGAACATGCTGATTCCTTCAGTGTAGCGCGCGTGCGGCCCAGGACATCTAAGGGCATCACAGACCTGTTATTGCTCAATCTCGTGCGGCTCGAAGCCGCCGGTCCCTCTAAGAAGAATTTTAATACGTCGCCAGTGAGTTGCGCGACCGAAGCCACGCACACCTAGATGGCGACGCCTATTTAGCAGGCTAGAGTCTCGTTCGTTACCGGAATTAACCAGACAAATCGCTCCACCAACTAAGAACGGCCATGCACCACCACCCACCGAATCAAGAAAGAGCTGTTAATCTGTCAATCCTTCCGGTGTCCGGGCCTGGTGAGATTTCCCGTGTTGAGTCAAATTAAGCCGCAGGCTCCACTCCTGGTGGTGCCCTTCCGTCAATTCCTTTAAGTTTCAGCTTTGCAACCATACTCCCCCCGGAGTCCAAAATCTTTGGTTTCCCGGAAGCTGCCCGCCGAGCCATTGTAGTAACGTCGGCGGATCGCTAGATGACATATTTACGGTTAGAACTAGGGCGGTATCTAATCGCCTTCGAACCTCTAACTTTCGTTCTTGATTGATGAAAACACCTTTGGCAAATGCTTTCGCTGATGTTCGTCTTGCGACGATCCAAGAATTTCACCTCTAACGTCGCAATACGAATGCCCCCAGTTATCCCTATTAATCATTACCTCGGAGTTCTGAAAACCAACAAAATAGAACCGAGATCATATTCTATTATTCCATGCACGAAATATTCAAGCAGCATTTTGAGCCCGCTTTGAGCACTCTAATTTGTTCAAAGTAAAATTGTCGGCCCACCTCGACACCCGACAAAGAGCACCGCGATAGGATTTTGATATTGAACCGGCGTATTACCGCCGGCTCACCGACGATATGCTCCGCAGACGTGTCAGTATCACCGCGGATGCGGTGCACCGACAGCGCGGCGCACAAATGCAACTACGAGCTTTTTAACCGCAACAATTTTAGTATACGCTATTGGAGCTGGAATTACCGCGGCTGCTGGCACCAGACTTGCCCTCCAATTGTTCCTCGTTAAAATATTTAAAGTGTACTCATTCCGATTACGAGGCCTCGTAAGAGTCCCGTATCGTTATTTTTCGTCACTACCTCCCCGTGCCGGGAGTGGGTAATTTGCGCGCCTGCTGCCTTCCTTGGATGTGGTAGCCGTTTCTCAGGCTCCCTCTCCGGAATCGAACCCTGATTCCCCGTTACCCGTGACAACCATGGTAGTCGCAGAAACTACCATCGAAAGTTGATAAGGCAGACATTTGAAAGATGCGTCGCCGGTACTGGACCATGCGATCGGCAAAAGTTATCCAGATTCATCAAAATTAACGACTTCGAACGCGAGGCTCTCCGTCGATTGGTTTTGATCTAATAAAAGCACTCATCCCATCACTGGTCAGAGTTCTGATTGCATGTATTAGCTCTAGAATTACCACAGTTATCCAAGTAACTGAGTAAGATCTAAGGAACCAAAACTGATATATTGAGCCATTCGCGGTATCGCCTTAATACGGCTTGCACTGAGACATGCATGGCTTAATCTTTGAGACAAGCATATAACTACTGGCAGGATCAACCAGGGA

>*28S ribosomal RNA* (*28S*)

AAAAAAACACGCCACATCGACATAAGATTTCTCCTTGAGCTTAGGATCGACTGACTCGCGAGCAACTACTGTTCACGCGAAACCCTTCTCCACGTCAGTCCTCCAGGGCCTCGCTGGAGTATTTGCTACTACCACCAAGATCTGCACCGACGGAGGCTCCAAGCGGGCTCACGCCCAGACCCTTCTGCGCACTCCGCCGCGCACGTCCTACTCGTTACGGCTTAATGACGTCACAATAAAGAACGTCGCACATGCCCGTAACGGTAGTGTATAGGCAAAACGCTTCAGCGCCATCCATTTTCAGGGCTGGTTGCTTCGGCAGGTGAGTCGTTGCACACTCCTTAGCGGATTCCGACTTCCATGGCCACCGTCCTGCTGTCATGAGCGACCAACGCCTTTCATGGTGTCCCATGAGCGTTTTTTAGGCGCCTTAACACTACGTTTGGTTCATCCCACAGCGCCAGTTCTGCTTACCAAAATTGGCCCACTTGGCACCGTCATCAGATCTCCG

>*Actin* (*ACT*)

AGAGGGAAATCGTGCGTGACATTAAGGAGAAGCTGTGCTATGTCGCCCTGGACTTCGAGCAGGAAATGGCCACCGCTGCCGCCTCCACCTCCCTCGAGAAGTCCTATGAACTTCCCGACGGTCAGGTCATCACCATCGGTAACGAGAGGTTCCGTTGCCCTGAAGCCCTCTTCCAGCCTTCCTTCTTGGGTATGG

>*Arginine kinase* (*AK*)

GAAAAGCCGCAGCAATGGTGGACGCCGCAACCATGGAGAAATTGGAGGCTGGTTTCAGCAAGCTCGCTGCCTCTGATTCGAAGTCGCTGTTGAAGAAATACCTTACCAGGGAGGTCTTTGACGCTCTTAAAAACAAGAAAACATCCTTTGGATCAACATTGTTGGATTGCGTCCAGTCTGGTTTTGAAAACCACGACTCTGGTGTTGGAATCTACGCCCCTGATGCTGAGGCGTACACAGTCTTTGCTGATCTGTTCGACCCCATCATCGAAGACTACCACAATGGTTTCAAGAAAACCGACAAGCACCCACCCAAGAACTGGGGTGATGTCGAGACTTTTGGACAACTGGATGCCGCTGGTGAATTCATCATTTCCACCCGTGTGCGTTGCGGTCGCTCTATGGAGGGCTACCCCTTCAACCCCTGCTTGACTGAGGCTCAATACAAGGAGATGGAAGAAAAAGTCTCTAGCACTCTCTCTGGCCTAGAAGGTGAACTCAAGGGTACCTTCTACCCTCTCACTGGCATGTCCAAGGAGGTACAACAGCAGCTGATCGATGACCACTTCTTGTTCAAAGAGGGTGACCGTTTCCTCCAGGCCGCTAATGCTTGCCGTTTCTGGCCCACTGGCCGTGGTATCTACCACAATGAGAACAAGAGTTTCTTGGTATGGTGCAATGAGGAAGACCATCTTCGTCTTATTTCCATGCAGATGGGTGGTGACTTGAAACAGGTATACAAGAGGCTGGTTACAGCTGTGAATGATATTGAGAAGAGGATTCCCTTCTCTCACGATGACAGGCTTGGTTTCTTGACCTTCTGCCCCACCAATCTTGGTACTACAGTCCGTGCGTCTGTCCATATCAAGCTGCCTAAACTGGCCGCAGACAAGGCAAAGCTTGAGGAAGTCGCATCTAAATACCACTTGCAGGTACGCGGAACGCGCGGAGAGCACACAGAGGCTGAAGGCGGTGTCTATGACATCTCCAACAAGAGGCGTATGGGTCTCACCGAGTACGATGCCGTCAAGGAAATGTACGATGGCATCGCTGAACTGATTAAGATTGAAAAATCGCTGTA

>CyclinA (CYCA)

TTTACAAGATAAGAAAAATGTGGTCGAATCTCCTATGTCAGTTGTGGACACAAGTGTGCTTTCTATGTCTGTATCTAAGAATGAAAGTCAGATTATTGAGGATGAAGACACAACCACTGCACAGACTGATCGGGAATTATTCTTCCATGTCATAGAATACAGGCAAGACATCTATGAATACATGAAGGAAATAGAAGTGAAGAATAGAGCCAACCCCCGCTACATGCGTAAGCAGCCAGATATTACACATATGATGCGTTCTATTTTGATTGACTGGCTGGTTGAAGTGTGTGATGAATACGGTCAACAAAGTGAGACATTACATTTAGCTGTGTCATATGTAGACCGCTTCTTGTCTTATATGAGTGTTGTTCGTACTAAGTTGCAGCTTGTGGGCACAGCCGCTACTTACATTGCAGCAAAATATGAGGAAGTGTATCCACCAGAAGTATCAGAGTTTGTGTACATCACTGACGACACATACACGAAACGTGAAGTCTTGAGAATGGAACATTTGATTCTGAAGGTGCTGTCCTTCGACCTGTCTACGCCAACGTCTCTTGCTTTCTTGTCACTTTACTGTATATCAAATGGACTTTCAAAGAAGACCTTTCACTTAGCTTCTTACCTTGCCGAACTCTGCCTTTTGGAAGCAGATCCTTACCTCCAGTTTAAGCCATCAATCATAGC

>*Elongation fator 1 alpha* (*EF1A*)

ATGGGCAAGGAAAAGGTTCACATTAACATTGTCGTCATTGGACACGTCGACTCAGGCAAGTCCACAACCACTGGTCACTTGATCTACAAATGTGGTGGTATCGACAAACGTACCATCGAAAAGTTCGAGAAGGAGGCCCAGGAAATGGGTAAAGGTTCGTTCAAATACGCCTGGGTATTGGACAAACTAAAGGCTGAGCGTGAACGTGGTATCACTATTGACATCGCTTTGTGGAAGTTCGAAACCAGCAAATACTATGTCACCATCATCGACGCCCCCGGACACAGAGATTTCATCAAGAACATGATCACTGGTACTTCCCAGGCTGACTGCGCTGTGCTTATTGTAGCTGCTGGTACTGGTGAGTTTGAGGCTGGTATCTCTAAGAACGGACAAACCCGTGAGCATGCTTTGCTTGCTTTCACACTTGGTGTCAAGCAGCTCATTGTTGGTGTTAACAAAATGGATTCCACTGAGCCCCCATACAGCGAATCTCGTTTCGAGGAAATCAAAAAGGAAGTCTCCTCTTACATCAAGAAAATTGGTTACAATCCAGCTGCTGTCGCTTTTGTACCCATTTCTGGCTGGCATGGAGACAACATGTTGGAGCCCTCTACCAAAATGCCCTGGTTCAAGGGATGGATGGTTGAGCGCAAGGAAGGCAAAGCTGAAGGCAAATGCCTCATTGAGGCCCTGGACGCCATCCTGCCACCTGCTCGTCCCACAGACAAGCCCCTCCGTCTTCCTCTCCAGGACGTATACAAAATCGGCGGTATTGGTACAGTGCCAGTAGGTCGTGTTGAAACTGGTATCCTCAAGCCTGGTACAATTGTTGTCTTCGCACCTGCCAACATCACCACTGAAGTCAAATCTGTGGAGATGCACCACGAAGCCCTGCAAGAGGCAGTACCCGGTGACAACGTAGGTTTTAACGTAAAGAACGTATCTGTCAAGGAATTGCGCCGTGGCTACGTAGCTGGTGACTCTAAGAACAACCCACCTAAAGGTGCCGCCGATTTCACAGCACAGGTTATCGTACTGAACCACCCTGGTCAAATTTCAAACGGTTACACGCCTGTGTTGGATTGCCACACAGCTCACATCGCTTGCAAATTCGCCGAAATCAAAGAAAAGGTTGACCGTCGTACTGGTAAATCCACTGAGGAAAATCCAAAATCCATCAAATCTGGTGATGCTGCCATTGTTAATCTAGTTCCATCCAAGCCTCTATGTGTAGAGTCTTTCCAGGAGTTCCCTCCCCTCGGTCGTTTCGCTGTGCGTGACATGAGGCAAACAGTTGCTGTAGGAGTAATTAAGGCTGTAAACTTCAAGGATGCCTCCGGTGGTAAGACAACCAAAGCAGCTGAAAAGGCCGCCAAAGGCAAGAAGTA

>*Glyceradehyde-3-phosphate dehydrogenase* (*GAPDH*)

TCAGGAGCTACCCAACGTTACTTTAGTCTGCTCTGCTGCTTGCTTTGCTTGTCCTTCTAGCGACATTGTCTTGCCTTGTCACATTGTTAAACTCTCATCTCGTTGGGAGCGTACGTGCAGTAGTCTGTTATTTATTTGTTGCTCACATATTTAATCCGCAATGTCGAAAATTGGTATCAATGGATTTGGCCGCATCGGGCGTCTGGTTCTTCGTGCAGCCGTGGACAAAGGTGCTCAGGTAGTTGCCATCAATGATCCCTTCATTGGTCTGGACTACATGGTTTACCTTTTCAAATATGACTCCACTCATGGTCGTTTCAAGGGAACGGTTGAAGTTCAAGATGGCTTCCTTGTTGTCAATGGAAACAAAATCTCTGTCTTTTCTGAGAGAGATCCTAAAGCCATTCCGTGGGGCAAGGCTGGTGCAGAATATATCGTGGAATCTACTGGTGTATTCACAACCACAGAAAAAGCATCCGCTCATTTAGAAGGTGGTGCCAAGAAAGTCATCATCTCTGCTCCTAGTGCTGATGCTCCCATGTTCGTGGTTGGAGTAAACCTCGAAGCCTATGATCCCTCTTACAAGGTTATCTCTAACGCTTCTTGCACTACAAACTGCTTGGCTCCTCTTGCAAAGGTTATCCACGACAATTTCGAAATTGTTGAAGGTCTCATGACCACTGTACACGCAACCACTGCCACTCAAAAAACCGTCGATGGACCCTCTGGCAAACTGTGGCGTGATGGCCGTGGTGCTCAACAGAACATAATCCCCGCCTCCACTGGCGCTGCTAAGGCTGTGGGTAAAGTCATTCCTGCCCTAAATGGAAAACTGACCGGTATGGCCTTCCGTGTACCTGTTGCTAACGTCTCTGTCGTCGATCTCACAGTCCGCCTAGGCAAGCCTGCGTCTTATGACGCTATCAAACAGAAGGTTAAGGAGGCTGCCCAGGGCCCATTGAAGGGTATTCTTGATTACACTGAGGAACAGGTTGTATCTTCAGACTTCATCGGTGACACACACTCTTCAATCTTCGATGCTGCTGCCGGCATCTCTCTCAATGAGAACTTCGTCAAGCTCATCAGTTGGTACGACAATGAGTTTGGTTATTCCAACCGTGTCATCGATCTCATCAAGTACATCCAAACCAAGGATTAAATCTTGAAAGAAATGTAAGACATTAATGTTGTTATGAATGATATATGATTATTATTTGTAGAGTTTTGTAATAATTTAAGCTAAGTTTAACAGTCACCCTAAATTTATTTAACTACTTTGATAGTAGAGCTTTGATAAGTTTATATTATCACATTAAGATTGATGCAGTATGCAAATAAACATGTATGTAATCTAAAAAAA

>*Ribosomal protein L10* (*RPL10*)

TTCCTCCATGCATCAAGGGGTCCATGCTCAGGGCGATACCTGACATTGCATCCGTCGTTGGCGAGACGGCCATCCTCACGCAACTTTTCAAATTCGTCACGGTCATACTTAGTGAAACCCCATCTCTTAGAGACGTAGATCTTCTGGCGACCAGGGAACTTGAACTTGGCACGACGCAGAGCCTCAATGACCTGCGCCTTCCAACGGTCACTAGAGCGCACAGACATGATGGGTTGTCCAATGCGTACTCTCGCTACAGTACCCTGAGGCTTGCCGAACGCGCCACGCATCCCAGTCTGGAGCCTATCAGCTCCAGCGCACGATAACATTTTGTTGATGCGGATAACATGGAATGGGTGCAGCCTCATTCTAATGTGGAACTGATCTTTGCCACAGTTCTTCACAAGATACTTGTTGCAGCAAATACGCCCTGCTTCCAAAGCTTCAGAGCTGAGCTGCTCATACTCGTCAGACACAAGGTGCACGCATAGTGGGAAGTCATCAACGGGTGCCCTCTTCTTACCCAAGTCGAAGATACGGATCTTAGGGTCGGGTACACCACGACAGAACCGAGATTTAGGATACGGTTTGTTTTTACAGTACCGGTAACATCTTGCTGGCCGGCGCCCCATTGTGATCTTCACAATGT

>*Ribosomal protein L27* (*RPL27*)

TTTTTTTTTTCATTACGCAAGTAATGTATTTTAAAATAACTTAGAACCTAAGCTTCTGGAAGAACCACTTGTTTTTCCCACTCTTGTATCTTTCCTCAAAGCGCACGCGTGTGTTGAAGCGTAGCTTCTTGCGTTTTGCGGGATCTTTTAGGTCCTTAGCGCTGAACTTTTCAAAGCTGAAGTCTACTGAGTAACGGGTGGGCATTAAGTGATTGTAGTTTACTACCTTCACGAAGGGCTTGACTTTTGAGCGCTTGTGGATTTTGTTTTTACCCATCCTCTTGTGTACTTTGCGGGGATATCTGTCAATGCCGGCGACGAAAGCGTGACCGTAAGGTTTTTCTGATGTGCCCTCATCGTAATTTTTAACAACGATGGCCTTACGCCCTGCGTACCGGCCGCTGAGGACCAGCACTACCTTACCTGGCTTCATAATCTTACCCATTTTGGGTGGATATTCCGTTTA

>*Ribosomal protein L28* (*RPL28*)

TTATAACTTATTCTGTCTTAGCTGCTGCCGCTTTGGGTTTCTTCGCCTTGATGGGCCTTTGTGAGCGAAGGATAGCAGAAGCACGGCGGAGGGTAGCCTTGGACAAATCAGTGCGGTAATGGTTAGCCTTTAACAACCTCTTCACTTTGAATAGCGATCTCCTGGCACCAGCTTTGAATGTACGACGTACGGAGTTTTTGGCTGGTTTGTTAGTCGCCTTAGCCTTCTTGTATACAACTGTAAAACCTTTCCTGTCAGGATTTTCTACAACACCAACAGCCTTCTTCTGGATCAAGCCGTTGTATCTGTAAGAGTTGAGGTTAGTCACATTGTTCGGTTCCTTGCTGAAGGGTTTCTTGATATTACGCTTTTTCACTAGGAAAGCGTTGTTGTTGCGAATGATCATCCAGTTTAAATGAGACGACATTTTTGTGGTT

>*Ribosomal protein S2* (*RPS2*)

GCTGAAGTATAGCAGTCCTGAACACCAGCCATCTGAAGTAGCTTCTTAGGGACTGGAGCAGACACAATACCAGTACCACGAGGAGCAGGGATCAATCTTACAGTGACAGAACCACATTTGCCCGTGACCTTGCATGGCACAGTGTGGGGTTTGCCAATCTTGTTACCCCAGTATCCTCTGCGAACAGGCAAAACTGAGAGCTTGGCAAGTATGATAGCCCCACGGATAGCTGTAGCAACTTCTTTGCTGCATTTGACACCCAAACCGATGTGTCCATTGTTGTCACCAATGGCAACAAAAGCCTTGAAGCGGGTACGCTGACCGGCACGGGTCTGCTTCTGCACGGGCATAATCTTCAGTACCTCATCATTCAGGGACGGTCCAAGGAAAAAGTCGATGATTTCGAACTCTTTGATGGGCAAAGAGAAAAGATAGATGCTCTCAAGTTTGTCAATCTTTCCTTCACGGACTAGACGACCAAGTTTAGTTACAGGTACCCAGTCCTTAGGGTCTTCCTTTCCACGACCACGGCCGCGACCACGACCGCGTCCACGTCCACGAGGTCCGCCACGGCCCCTATCGCCGCCACGGGATCCAAAACCACCGCGGAAACCGCCACGTCCACCGGCTGGAGCTGCGTCCGCCATTGTTGAA

>*Ribosomal protein S13* (*RPS13*)

TTAGTTTATTTTAACTTAAGCCACCAAAGCGGAAGCCGTGCTTGACTCATATTTCCAGTTAGGCGGGAGCACACTCTTTGTCTTGTAGTAGCGAGCTAGTCTGTGAATTCTAGACTCAACAAGAATCAGTCTGAATTTGCTGTCCTTGTCCTTTCTATTACGCTCCAAATGTTTCCTCATGGCTACAGCCTTCTTGATCAAGTAGTACAGATCCTCGGGTAGGTCAGGTGCCAGACCCATAGCTTTCATGATACGGAGGATCTTTTTGCCAGTTACAAATCTGACTTGTGCTACTCCATGGGAATCCCTGAGTACAACACCAATTTGCGAGGGAGTAAGACCCTTTTTACCAAGTTTGAATATCTGTTCCTTGACATCATCAGCAGTCAGTTTTAACCAGGTAGGGACACTGCGACGGTATGGCAGCGCTGATTGGGAAATACCCTTACCAGGTGCGTGCATACGACCCATGGTTGCGGCTTTTTATAAAAATCGCCGATCTGACA

>*Ribosomal protein S15* (*RPS15*)

TTCTGTCAATCAAATCGGAAACATGGCTGAGGTTGATGAAACTCTCAAGAAAAAACGTACCTTCAGGAAGTTTACCTTCCGTGGTGTTGATCTTGATCAGCTTCTTGATATGCCCAATGAGCAACTCATGGAGTTGATGCATGCGCGCGCTCGCAGGAGGTTCGCCCGTGGTCTTAAACGCAAACCTATGGCTTTAGTGAAGAAACTCCGTCGTGCAAAAAAAGAGGCCCCACCAAATGAGAAGCCCGAGATAGTAAAGACTCACTTGAGAAACATGATAATTGTTCCTGAGATGGTTGGCTCTATTGTAGGTATCTACAATGGAAAGACCTTCAATCAGGTTGAAATCAAACCGGAAATGATTGGACATTATCTTGGTGAATTCTCAGTCACATACAAGCCTGTGAAACACGGTAGGCCTGGTATTGGTGCCACCCACAGCTCCAGGTTCATTCCACTGAAGTA

>*Troponin C* (*TPNC*)

GGGCAGTCGTGTTGTGCGTCTCATAGTGTTAACTAGTGACATCAAACATGGTTCTGGATACAGAAGACTGGGCCGATGAGCTCCCACCAGAACAGATTGCCGTTCTTCGCAAGGCGTTTGATGGCTTTGACCACAACCGCTCAGGAAGCATCCCGTGCGATTTTGTCGCAGATATCCTTCGGATGATGGGGCAACCATTTAACAAGAAGATTCTCGAAGAACTTATAGAGGAAGTTGACGCTGACAAGTCAGGCCGCCTGGAATTCGGTGAGTTCGTAACACTGGCTGCCAAGTTCATCGTAGAAGAAGACGCGGAAGCTATGCAGAAGGAGCTGAGGGAAGCCTTCAGATTATACGACAAAGAAGGCAACGGTTACATTCCCACATCAAGCTTACGCGAGATCCTGCGAGAATTGGACGAGCAGCTCACAGAAGAAGAACTCGACGGACTTATCCAGGAAATTGATACTGACGGAAGCGGCACCGTGGACTTCGATGAGTTCATGGAGATGATGACGGGAGAATAAATGTCT

>*α-Tubulin* (*α-TUB*)

TTTTTTTATTTTATGTCACCGGAATGTACTCGGTGCGAGTGTACCGTACTTGTTGAACAGTTTAGTACTCTTCGGCTCCCTCTCCTTCGCCTTCAGCGGAGTCCATGCCGACTTCTTCGTAATCCTTCTCCAGAGCGGCCAAGTCCTCACGAGCCTCTGAGAACTCACCCTCCTCCATACCCTCACCGACGTACCAGTGCACGAAGGCACGCTTAGCGTACATGAGGTCGAATTTGTGGTCCAGGCGAGCCCAGGCCTCTGCGATGGCGGTTGTGTTGGACAACATGCAGACGGCGCGCTGTACCTTGGCCAAGTCACCACCAGGTACGACAGTCGGGGGCTGGTAGTTGATACCGACCTTGAAACCGGTGGGACACCAGTCTACAAACTGGATGGTCCTCTTAGTCTTGATAGTGGCGATAGCCGCATTCACATCCTTTGGCACAACGTCACCACGGTATAACATGCAGCAGGCCATGTATTTACCATGACGTGGGTCACATTTGACCATCTGGTTGGCGGGCTCGAAGCACGCGTTAGTGATTTCAGCCACAGACAGCTGTTCGTGGTATGCCTTCTCGGCAGAAATGACGGGTGCGTATGTCACCAGAGGGAAGTGGATACGTGGGTATGGCACCAAGTTGGTCTGGAACTCTGTAAGATCGACATTGAGAGCGCCATCGAATCGTAGGGAAGCAGTGATAGAGGACACGATTTGGCCGATGAGACGATTCAGGTTGGTGTAAGTGGGGCGTTCGATGTCAAGGTTGCGGCGGCAGATGTCGTAGATAGCTTCATTGTCAACCATGAAAGCACAGTCAGAGTGCTCCAGAGTGGTGTGGGTGGTGAGGATAGAGTTGTAGGGCTCCACGACGGCGGTGGACACCTGCGGCGCAGGGTAGATGGCGAACTCCAGCTTGGACTTCTTGCCGTAGTCCACGGAGAGTCGCTCCATGAGGAGGGATGTGAACCCTGAGCCAGTGCCTCCACCGAACGAGTGGAAGATTAGGAAGCCCTGGAGGCCTGTGCATTGGTCGGCCAGTTTACGGATGCGATCAAGTACCAGGTCTACGATTTCCTTGCCAATGGTGTAGTGGCCACGGGCATAGTTGTTGGCCGCATCTTCTTTACCAGTGATAAGTTGTTCTGGATGAAACAACTGTCTGTATGTGCCGGTGCGGACCTCATCAACTACTGTTGGTTCCAAGTCAACAAACACAGCCCTGGGGACATGTTTGCCTGCACCGGTCTCACTGAAGAATGTGTTGAAGGAGTCATCGCCACCACCCACGGTCTTGTCTGTGGGCATCTGGCCATCAGGTTGGATGCCATGCTCAAGGCAGTATAACTCCCAGCAGGCATTACCGATCTGGACTCCAGCCTGGCCAACATGTACGGAGATGCACTCACGCATTTTGTTTGGAGTTTTGTGAACTTAACGTACAAATAAATTAATTAAATAATTCAGTCGTCGTTCAG

>*β-actin* (*β-ACT*)

ACTCGCAGTCTCGCTGCCGTAGAGTCAACATCCACCGCGTCCCAAAACACACACACACGCCAAAATGTGCGACGACGATGTTGCTGCGCTTGTAGTCGACAATGGCTCCGGCATGTGCAAAGCCGGTTTCGCCGGCGATGACGCGCCCCGCGCCGTCTTCCCGTCCATCGTGGGTCGCCCCCGTCATCAGGGTGTGATGGTCGGTATGGGCCAGAAAGACTCCTATGTAGGCGATGAGGCCCAGAGCAAGAGAGGTATCCTCACTCTCAAATACCCCATCGAGCACGGTATCATCACCAACTGGGATGACATGGAGAAGATCTGGCACCACACCTTCTACAACGAGCTGCGCGTCGCCCCTGAGGAGCACCCAGTCCTCCTGACTGAGGCCCCCCTCAACCCTAAGGCCAACAGGGAGAAGATGACCCAGATCATGTTTGAAACCTTCAACTCCCCGGCTATGTACGTCGCTATCCAGGCCGTGCTTTCCCTGTACGCTTCCGGTCGTACCACCGGTATCGTACTTGACTCCGGTGATGGTGTCTCCCACACTGTGCCCATCTACGAAGGTTACGCTCTACCCCATGCCATCCTCCGTTTGGACTTGGCTGGCCGTGACTTGACCGACTACCTCATGAAGATCCTCACCGAGAGGGGTTATTCTTTCACAACCACCGCTGAGAGGGAAATTGTCCGTGACATTAAGGAGAAGCTGTGCTATGTCGCCCTGGACTTCGAGCAGGAAATGGCCACCGCTGCCGCCTCCACCTCCCTCGAGAAGTCCTATGAACTTCCCGACGGTCAGGTCATCACCATCGGTAACGAGAGGTTCCGTTGCCCTGAAGCCCTCTTCCAGCCTTCCTTCTTGGGTATGGAATCTTGCGGTATCCACGAGACCGTGTACAACTCCATCATGAAGTGCGACGTCGACATCCGTAAGGACTTGTACGCCAACACTGTCATGTCTGGTGGTACCACCATGTACCCTGGTATCGCCGACAGGATGCAGAAGGAGATCACAGCCCTCGCTCCCTCCACAATCAAGATCAAGATCATCGCTCCCCCTGAGAGGAAATACTCCGTATGGATCGGTGGATCCATCCTGGCTTCCCTCTCCACCTTCCAACAGATGTGGATCTCCAAGGAGGAATACGACGAGTCTGGCCCTGGCATTGTCCACCGCAAGTGCTTCTAA

>*β-Tubulin* (*β-TUB*)

TAAATAAATTACAATGAGGGAAATCGTGCATATTCAGGCTGGACAGTGCGGAAACCAGATTGGAGCCAAGTTCTGGGAGATCATCTCCGATGAGCATGGTATCGACCCCACCGGCGCCTACCATGGTGACTCGGACTTGCAGCTGGAGCGCATCAACGTATACTACAATGAAGCCTCCGGCGGCAAATACGTGCCCCGCGCCATCCTCGTCGATCTGGAGCCCGGCACTATGGACTCTGTGCGCTCAGGACCCTTCGGACAGATCTTCAGACCCGATAACTTCGTATTCGGACAGTCTGGTGCCGGCAACAACTGGGCCAAAGGTCACTACACGGAGGGCGCTGAGCTCGTAGATTCAGTATTAGATGTCGTACGCAAAGAAGCAGAGTCATGCGATTGTTTACAAGGATTCCAACTTACACACTCGCTCGGCGGTGGTACCGGATCCGGTATGGGCACACTTCTTATCTCCAAAATTAGAGAGGAATATCCCGATAGAATTATGAACACATATTCAGTTGTACCTTCGCCAAAAGTGTCAGATACAGTAGTAGAACCATACAATGCAACACTATCAGTACATCAGTTAGTAGAAAACACAGACGAAACCTACTGTATCGACAACGAGGCTCTCTATGACATCTGCTTCCGCACGCTCAAACTGTCCACACCCACGTACGGCGACCTCAACCACCTGGTGTCGCTCACAATGTCGGGCGTGACAACGTGTCTGCGGTTCCCCGGCCAGCTGAATGCGGATCTCCGCAAGCTGGCCGTCAACATGGTGCCGTTCCCTCGTCTGCACTTCTTCATGCCCGGCTTCGCGCCGCTAACGTCCCGGGGCAGCCAGCAGTACCGCGCGCTCACCGTGCCCGAGCTCACCCAACAGATGTTCGACGCCAAGAACATGATGGCGGCCTGCGACCCGCGCCACGGACGCTACCTAACAGTCGCCGCCATCTTCCGTGGACGCATGTCCATGAAGGAGGTCGACGAACAAATGCTGTCGATCCAAAACAAAAACAGCAGCTTCTTCGTAGAATGGATTCCTAACAATGTTAAAACTGCTGTCTGCGATATTCCTCCTAAGGGTCTGAAGATGTCTTCTACCTTCATCGGTAATACCACAGCCATCCAGGAGCTGTTCAAGAGAATATCAGAGCAATTCAGCGCTATGTTCAGACGTAAAGCCTTCTTGCATTGGTATACTGGTGAGGGTATGGACGAAATGGAATTCAATGAAGCTGAAAGCAACGTTAACGACTTGGTATCTGAATACCAGCAGTATCAGGAAGCAACCG

>*Heat shock protein 70* (*HSP70*)

CAAAGATGCCAGCGATCGGTATTGACCTTGGAACTACGTATTCGTGCGTCGGAGTGTGGCAGCACGGCAACGTGGAGATCATCGCCAACGACCAGGGCAACCGCACCACGCCGTCCTATGTGGCGTTCACGGACACCGAGCGTCTTATCGGAGATGCAGCCAAGAACCAGGTCGCCCTGAACCCCAACAACACAGTCTTCGACGCCAAACGTCTCATCGGAAGGAAATTCGACGACCCTAAGATTCAGCAGGACATGAAACACTGGCCCTTCAAAGTGATCAACGACTGCAGCAAGCCCAAGATCCAGGTGGAGTTCAAGGGCGAGACGAAGCGCTTCGCGCCCGAGGAGATCAGCAGCATGGTGCTGAGCAAGATGAAGGAGACGGCGGAGGCGTACCTGGGCGCGGCGGTGCGCGACGCCGTCATCACGGTGCCCGCGTACTTCAACGACTCGCAGCGGCAGGCCACCAAGGACGCGGGCGCCATCGCCGGCCTGAACGTGCTGCGCATCATCAACGAGCCCACGGCCGCCGCGCTGGCCTACGGGCTGGACAAGAACCTCAAGGGCGAGCGCAACGTGCTCATCTTCGACCTGGGCGGCGGCACCTTCGACGTCTCCATACTCACCATCGACGAGGGCTCGCTGTTCGAGGTGAAGGCCACGGCCGGCGACACGCACCTGGGCGGCGAGGACTTCGACAACCGCCTGGTGAACCATCTGGCGGACGAGTTCAAGCGCAAGTACAAGAAGGACCTGCGCACTAATGCGCGCGCCCTGCGCCGCCTGAGGACGGCCGCCGAGCGCGCCAAGAGGACGCTTTCCTCCAGCACCGAGGCCACCATCGAGATCGACGCGCTCTACGAGGGCATGGACTTCTACACCAAGGTGTCCCGCGCGCGCTTCGAGGAGCTGTGCTCGGACCTGTTCCGCGGCACGCTCGACCCCGTGGAGAAGGCGCTCAAGGACGCCAAGCTCGACAAGGGCCAGATCCACGACGTGGTGCTCGTGGGCGGCTCCACGCGCATCCCCAAGGTGCAGAGCCTGCTGCAGAACTTCTTCTGCGGCAAGAAGCTCAACTTGTCCATCAACCCCGACGAAGCGGTGGCGTACGGCGCGGCCGTGCAGGCGGCCATCCTCAGCGGCGAGCAGGACTCCAAGATCCAGGACGTGCTGCTGGTGGACGTGGCGCCCCTGTCGCTCGGCATCGAGACCGCCGGCGGCGTCATGACCAAGATCATCGAGCGCAACTCCAAGATCCCGTGCAAGCAGACGCAGACGTTTACCACGTACTCTGACAACCAGCCGGCCGTCACCATCCAGGTGTACGAGGGCGAGCGCGCCATGACCAAGGACAACAACCTGCTGGGCACGTTCGACCTGACGGGCATCCCGCCCGCGCCGCGCGGCGTGCCCAAGATCGACGTGACGTTCGACATGGACGCCAACGGCATCCTGAACGTGTCCGCCAAGGAGAACAGCACGGGCCGCAGCAAGAACATCGTGATCAAGAACGACCGCGGACGCCTGTCGCAGGCCGAGATCGAGCGCATGCTGTCCGAGGCGGAGCGCTACAAGGAGGAAGACGAGCGGCAGCGGCAGCGTGTGGTGGCGCGCAACCAGCTCGAGGCGTACGTGTTCGGCGTGCGGCAGGCGCTGGACGAGGCGGGCGACAAGCTGCCGCAGCACGAGGCAGCCGCGGCTCGCAGCGCCTGCGACGACGCGCTGCGCTGGCTCGACAACAACTCGCTAGCCGAGCGCGACGAGTACGAGCACAAACTGCAAGAGCTGCAGCGCCAGTGCGCGCCGCTCATGACCAAGCTGCACGGCGGGGCG

>*Heat shock protein 90* (*HSP90*)

TTAATCTACTTCCTCCATGCGTGAAGCCTCATCAGCATCACCCTCTAGTGGTGGGACATCTCCAGCACTCGCTTCCTCGACCTGGATAGGCTCGTCTTCATCAATGCCAAGACCAAGCTTGATCATGCGGTAGATGCGAGATGCATGCACCTGGGGCTCATCTAGAGCAAAACCAGAAGACAACAGAGCAGTCTCATAGAGCAAGATAACAAGGTCCTTAACAGCCTTGTCATTTTTGTCTGCCTCAGCCTTCTGCCTCAGTGTCTCAACAATGGAATGATCAGGGTTGATCTCAAGATGTTTCTTCGCTGCCATGTAGCCCATGGTGGAAGTATCACGGAGTGCTTGTGCCTTCATGATACGTTCCATGTTAGCAGACCAACCATACTGAGCAGTGACAATACAGCAAGGTGATTCTACTAGACGGTTTGATACAACAACTTTCTCTACTTTGTTGTCCAGGATGTTCTTCATGACCTTGCACAGACCTTCAAACTTAACCTTGTCTTCCTCACGCTTCTTAATCTCTTCCTCATCCTCTGGAAGTTCAAGACCTTCCTTTGTGACAGACACCAAGGTCTTGCCATCATATTCTTTCATCTGCTGTACTACGTACTCATCAATGGGCTCAGTCATGTAAACTACTTCAAATCCACGTTTCTTAACACGTTCTACAAAGGATGAGTTAGCCACCTGGTCACGGTTCTCACCAGTAATGTAATAAATGTGCTTCTGGTTTTCCTTCATGCGGGAGACATACTCTTTCAGTGAGCATGCCTCATCACCAGACGCAGATGTGTGGTAACGGAGCAAATCTGCCAGTTTAGACCTATTCTGAGAGTCTTCATGGATACCTAATTTTAAGTTCTTGCTGAACTGTTCATAATACTTTTTGTAATTCTCTTTGTCTTCTGCCAATTCTTCAAACAATTCTAAGCATTTCTTCACCAAGTTCTTTCTGATTACTTTGAGGATTTTGTTCTGTTGGAGCATTTCACGAGAAATGTTGAGGGGCAAGTCTTCACTGTCAACAACACCCTTGATGAAGTTCAGGTATTCAGGAATAAGGTCTTCACAGTTGTCCATAATGAATACCCTTCTGACATACAGTTTAATGTTGTTCTTGCGCTTCTTGTTCTCAAAGAGATCAAATGGTGCTCTGCGAGGTACGAATAGAAGAGCTCTAAATTCTAGCTGACCTTCAACAGAGAAATGCTTCACAGCAAGATGGTCTTCCCAATCATTTGTGAGTGATTTGTAGAAGTCTCCATACTCTTCCTGTGTGATATCGTCAGCATTTCGCGTCCAGATAGGCTTGGTCTTGTTAAGCTCCTCATCTTCAGTGTATTTCTCTTTGATGGTCTTCTTTTTCTTCTTACTATCTTTCTTATCCTCATCGAGATCCTCGCCCACATCCTCAATCAATTTCTCATCCTCTTTTTCTTCCTCCGCTTCGTCATCAGACAATTCCTTCTCACGCTCCTTCTCAACTACTAATTTGATTGGATAGCCAATGAATTGAGAATGCTTCTTAACAATCTCTTTAATTTTGTGGTCTTCCAGGTACTCTGTTAAATCTTCTTTGATGTGAAGTACGATTTTGGTACCGCGTCCAAGTGGCTCGCCGTGGTCAGGGCGGATGGTGAATGAACCTCCCGCGGATGACTCCCACATGTACTGTTCATCATCGTTGTGTTTAGAGTGTACTGTCACGCGATCTGCCACGAGGTAACAAGAGTAGAAACCCACACCAAACTGACCGATCATGCTGATGTCGGCGCCGGCCTGCAACGCCTCCATGAATGCCTTAGTGCCTGATTTAGCGATGGTTCCGAGATTGTTCACCAAGTCTGCCTTTGTCATTCCAACACCGGTATCAATAATTGTCAATGTGCCTTCATTTTTGTTAGGAATAATCTTAATGTATAACTCCTTACCACTGTCGAGTTTTGACGGGTCAGTGAGAGACTCATACCGGATTTTGTCCAAAGCGTCCGATGAGTTGGAGATCAGCTCACGAAGGAAGATTTCTTTGTTTGAGTAGAATGTGTTGATGATGAGGGACATAAGTTGGGCGATCTCCGCCTGGAAGGCGAAGGTCTCCACCTCCCCAGATTCTGTTTGCATTTCTTCCGGCAT

>*Glucose-6-phosphate isomerase* (*GPI*)

ATTTCGGTGCCGCACTTTGAATTCATTAAATATAAATATGGAACCCAAAATTAATTTACAAAATGATCCTGCTTACATAGAAATTAAGAAATTTTACGAAGCGAACGCTTCTAAGATTAATATTCAACAATTATTCCAACAAGAAGCCGACCGTTTCAATAAATACAGCCTTCGCCTACCAACTCCAAATGATGGTGAGATCCTGTTGGACTACTCCAAGAACCGCATCAATGATGAACTGTGGAAGCTTCTACTTAATCTGGCCAAAAGCCGCAATGTGGAGAAGGCCAGAGATGCCATGTTTTCTGGAGAGAAAATCAATTTCACGGAAGACCGCGCTGTTCTGCACGTTGCTCTCCGCAACAGACAGAACCGTCCCATACTGGTAAATGATAAGGATGTGACTCCTGATGTCAACTCTGTGCTGGCACATATGAAAGAGTTCACCGGACAAGTCGTCAGTGGGCAATGGAAAGGTTACACCGGTAAACCAATTACGGATGTCATCAATATTGGTATTGGTGGGTCAGACCTGGGGCCACTGATGGTCACTGAGGCGCTCAAACCTTATGCCAATCATCTAAAGGTCCACTTCGTGTCCAACATCGACGGTACTCACTTGGCGGAGGTACTCAAGCGGCTAAAACCCGAGACGGCTTTGTTCATCATTGCGTCTAAGACATTCACCACGCAGGAGACTATCACCAACGCCACTTCGGCCAAAAACTGGTTCCTACAAGCAGCTAATGATCCATCAGCTGTAGCAAAGCACTTCGTGGCCTTATCTACAAATGCAGAGAAAGTAACTGCATTCGGCATCGACGCTAACAATATGTTTGGCTTCTGGGACTGGGTCGGTGGCAGGTACTCTCTGTGGTCTGCCATTGGTCTATCAATTTCCCTGTACATCGGCTACGAGAATTTCGAGAAACTTCTAGAAGGTGCCAATTATATGGACAACCATTTCACAACAGCACCGCTTGAGAAAAATGCTCCTGTGATCCTGGCTCTTCTAGGAGTGTGGTATTCCAACTTCTATGGTGCGGAGACACATGCCCTTCTGCCCTACGATCAATATTTACACAGGTTTGCAGCTTACTTCCAACAGGGTGATATGGAGAGTAACGGCAAGTACGTGACGCGTTCTGGGTCCTCAGTCCAATACAGTACGGGACCCATCGTGTGGGGAGAGCCCGGTACTAACGGACAACACGCTTTCTACCAACTCATACATCAAGGGACCAGACTAATTCCTTGCGACTTCATAGCACCAGCTCAGTCCCACAACCCCATCAGTAACGGCATCCACCACAAGATCCTACTCGCTAACTTCTTGGCACAGACTGAGGCACTTATGAAGGGCAAAACAGCAAATGAGGCTAAAGCAGAGTTAGAGAAGTCAGGCATGGCTGCAGATGCTATATCCAAGATACTGCCACACAAAGTGTTTGAGGGGAATCGTCCTACCAACTCCATTGTTGTAAAGAAAATAACACCATTCACATTGGGCGCTCTCATCGCTCTATATGAACACAAGATTTTCACTCAAGGCGTGATTTGGGATATCAACTCATTCGACCAATGGGGAGTAGAACTTGGCAAACAACTTGCTAAAGCTATTGAACCAGAACTGCAAGACAATTCTCCTGTGACGTCACACGATGCCTCCACTAACGGGCTCATTAACTTTCTCAAGGAAAATTTTTAAAAATTATGTTGCAATGTTAGGTATAGAGATGGGCGTAATTATTTAAATAAAAAATGTTAATTACAGATTTAAAAAAAAAACATTGTATAATATTAAAAAAAATATACATAAATATATATACTTTTGAAAGAAATTATTATTGAA

>*Hypoxia-inducible factors 1 alpha* (*HIF1A*)

GCTGGTTTCGTGCCTCAGTGGACCGATTAGTTGTGTTATTTAGCGAATGAAAGCATTCGGTGAAAATGTTTATCAATGGTCGGGGGGCGCAATATGACCAGGATTGCCCCGTTGTGGGCAATTTAGAAGACCAGGGATGGAATATGCAGTGGCCGATGAATGACCAATGGTCTAGACAACCGATTGTTAATCAGTGCAATTATGGTGATCCGAATTTCATGAGTCCCTCAGTGCAGTGCTACAGAAATCCGATGACGTATCAATATGAGCACAATTCGTTACGATATTATAATCCTCCTGTTCAAACGAATTACATTCCACCAGCTCCTATGATACAAAGCCTACCATGTCACCAACAGCAATCCTGGGATTACGATAACATGTGCTACAATATAGATGGCCAACCTTGTCAGTATACAACAGTAATTGATTTGGAAGATATGATGAATAATGAGAAACGTAAGGAGAAGTCGAGGGTTGCGGCTCGAACTAGACGCAATAGGGAGATGCACATCTTCGCAGAACTCTCAGCAGCCTTACCTGCCAGGAAAAAAGCCGTGGATCAACTCGACAAGGCTTCTGTTATGCGCCTGGCAATTTCTTACCTGAAGGTCAGGGATGTGGTTTCTTTATTGCCTCAAGTCGAAACTGAGCATCCCAAGATGAAGAATCCTGAAGGTATGGAGGAGGTGTCTTCGGAACTGTCGTATATGAAGGCTTTGGATGGCTTTGTGCTGGTGCTGTCCCAGCAAGGCGATATTGTTTACTGCAGCGACAATATTGCTGACCATCTTGGTGTTTCACAGATGGAGATCATGGGCCAGAGTGTATTTGAATTCAGCCATCCGTGTGATCACGACGAGATTCGTGACGCGTTGCGCTCTAGCGACGATGGACGCCGCGACTTGTTGCTTCGTATGAAATGCACCTTGACCAGCAAAGGACGGAACGTACATCTTAAATCTGCATCTTATAAGGTAATACACATCACAGGCCACTTGTTGAAACGTGATGAAATTATAGAAAAAAATAACAACGGAACCGAAGACAATGAAGTTAATAGTAAAGACAAGAAAGTCATCAAAGACGGCTCGCTTGTTGCCGTTGGCCGACCTATACCACATCCATCCAACATCGAAATACCACTGGACAGCAAAACGTTTCTCACAAAACACAGCCTTGATATGAAGTTCACAAACATTCACATCGACGAACCGTTGATGTCTACACTCGGTTTCGAGGCAGATGAATTAATCGGCAAATCGGTATATGATTACCATCATGCTGGCGATTCTGGCTCCCTAATTCATCAATTTAAATCATTATTCTCAAAAGGACAATGCGAGACTGGCCAGTACCGCTTCCTTGCCAAAACCGGAGGCTATGCCTGGATCCAGACACAAGCCACTGTCATCACTGACAAACAGCAGAAGCCGATCAGCGTCGTTTGCGTCAATTACGTTATCAGTGGCATCGAGTGCAAAGATGAAGTGTTTGCCGCACATCAGGTGCAACACGCCGATCTGAAGCCCACTGTGGCACCGATCGCGCCTGTGCCAGTCGTCCCGTCCGTTCAGATCGAACCACCTTCTAACGGTGCAATAGTCGCAGTCATACAACCTGAGGAGGAACGGCCCATACCTGTCACAGAATTTATATTCGCTCCGAGAAAGAAGGAAATGAATAAAGGCTTTCTCATGTTCTCCGAAGACGAGGGATATACAATGCTTAAAGACGAGCCGGAAGATCTGACACATTTGGCACCTACAGCTGGAGATGCTTGTATTCCTCTTATCGAGAACAGTCCGTTTGATATGTTTGATGACTTCATTTTGAATGATAACTATTGCAGTTTACTGGGTGATGATTTGGCTAATGACTCATCTGTTGGTTCTCTGACATCAGATCCTTTATTTTTGGCTTCACCAGAACCACAGGAAAACGACTCGTCATGTGAACGGTCGTCTTTAATAACAGACCTGTCGTTAGACACGTTTGACAGCAATAGGTCAGATAATGAAACAGATGATGGAAACTCCCCTTTTATCCCGTCAAATGACGAACTCCCGGTGTTGGAACCGGCAGTTATGTGGGGAGCATTGCCAGACAGTGTGAGCCTAGCTAGGCCACAGCGCACGGAACCTCAGACTATGACGTCAGCACCAGCTCTACAGCGTCTGCTAACAACGTCTTCTAAGGGACCGCAACCACAAGATCTCATTACAAATATATATTCAGACTCAGGCCTAATACCAAGCAGAGACGTTTCGAAGTGGGACACAGGAATAAAGAGAGTGCTAAAACAAGAGGAAGAGCCCTGTGCTAAACGTGTAAAGAGAAGCCCCTCACCGGTTCAGACGACACCAAGCAATCCATCTTCCAGCGTCCTCATGAACCTTCTGGATTTGCAACAGCAGTTATCACAAGCGGCGCCACAACAAATGGTTTCACAGCAACTGTGCACTCAGTACCAGATTGTCAGAAACCCACAACAAACAAATAATAACAAAATTAAGAGTAACATCCCTATAACAGTTATTAATATGTTACAAAACGGGCCTAAGCAAATAATACGTTCTAATACCCCTATTAATACGCAAATGTTTCGTTCAAATATGACTCCAAGTCCAATGTCACCATTGAGCTTGAACATAAGTCCTATGTATTCTTTACCATCCAGCCCAAACAGTTACAATAGCCCAGCAATAAGTCCTGCTAAAACAGAATGCATTATGAGTCCATATACGCCTCAATCCCTATCACCGGCTAGTACGTTCCAGCAATATAGCCCAACAAGCAGAATTATGTCTCCTGTAGGAATTATGAATGGATGTGACCCGTATCTTACCAATAAAATGCAGCCGAGTCCAGGATTTCTTCAGACGCCGGAAATACAATTAGACTCCGTGCCTCTCCCTAGTGACTTCTGGTCTGAGTCTGATATGATGCAAGGCACAAGCGACTTGCTTACTGCCCTTGATGATGTTAAATTAGTGTAAGAATATTTTAAGCATATATATTAATTAACATGTGAATTGATGATTGTAGAGTGATTTCTATATTTCCATTGGAACATTTATGGAGCGATTGAATAATGCAATATTTACATTTTATCAATAGAATGTTCAATTTTTCTCACAAACATTCCAATTGAAATGTTAGCTAAAATTTATTCAGCGGGAAATCACTCAGATAAATGCAGGGTCACGATAAAGTAATAACAATAACGAAGGGGTTCTACGCGACTTTTTTGTAACCAGCGTTGGTAAAATATTTCAATGTTTTAGTAGTAATACTTAATAAATAAATCTTTTGGGGTATATTGTATAGTATGTATACCAGCCAGTAACCCACAAATTATAGCCATCCTTTAATTTTATTAGAGACCTTGATAAGTGAATCCGGAAATTATTAAGAGTAGGAATTATTTTCTTTCGGCTAAACGGAGCTAAAAAGTAGAACAAAAGATTATCGAAACAGTAATACATCCATCTTCTAAACAATAAAAGGCAGGAACCTTGTTAGCGAGTTATATTATTAATTTACCAAAAAATCGATACTTTGTTTTATTCAATACAGGCGCTATGTAATCAATTCTCCAGAAGTTAATTTTAAAGTGAGGCATATAAAATCTTGATTTGCACATAGAAAAGTGGAATCATCAGACAGCTGATCTAGTGTCGAACACAAAGTCGATTGAACTACTTGAAATAATGTATTTTTCTCAATTGCCACTGAACTGTTAAAGACAGCCGTAGTTTATACTTTGTAGATATATTTCTGACATAGTTTACTAAGGCACAATGATTTTAAAATGAATTAACAAACAGGAAAGTAATTTATCTAGAATATTAACTTGTATACAGCATATGGTATTACATATTTTATATCGCTGTGGAAACGCACATCGCTTGGATTAATATTCACTATATAATTACACTGATGAGTGTCACTATTTGACCAGTGGCTGATACCTATGTATAGTACATACTATTGGTCTAATGAATGAGTCTTCGCATTTAATCAAAAGAATCTTTTTTGAATAAATCTTATATTCATTGCATTTTGGAGAATTAAACAGCGACAAAACAATATGCAATGATATTTCGTTACGCGTTGATTCCTTGCTATAACGCCAACACAAATTTAATTTATCTGTTAGTTGCATATCGTCTGGGTATATTTTATGAATGAGTGTGTGCAAACTATTTTAATGTAGTTTCAAAGGTGGCGCTGTAGACACATTTTTAAATTGTTTTCCTTTTTTCAAACAATCATTATGATTTTATACTTTGTACGAGTTTATATTTGAATTATTTGTTCAGATTATTAAATTGCATGTGTAATATATTAACTTGGTGCTTTTGATTTCCTTTACGCAATCTAAATTATATTATTCACAGCGCATGTGTCACTGTTGGGCACAGCCTATTCACTTGAGCAGTTTTATGATACCACGCTGGCAAGGTGACTTGGTTTGGCAGGTATAGGGGTTTGTTTTTAATTTCGATATACTTGGTCTTTTAACATGTCCTTTGTTTCGTACAGTACCCACGGCACGAAATAAATTGGTGGTTCATAGATATAAATAAATAATACAATATTAACATTTCCACATTTTTGCTAACCTCACCTACAGAGACGAATTTCATTTGTTTTTAAAATAGTCCACTTTCATTTCATTGAAAAAGCAAGGCAATAATCTGGTAATGATATTTAGGATCATGATATCAGAACAGTATATAATGTTTAAAAAAAGCATTGAAGTGCCCGCAGACTGTTATCTATTATTTATTCTGTGTTATGTTAATGATTTTGAAACAATTGTACAGATTAGACTGTTCAATTCATATATTGTTGTATTACATATTTCTTTTTTACTTTGTTTCCACTAAAACCAACACTCAAAAATATCAAAGCTGAAAATTTCTTACATCATTAATCGTCTGTAATATCGCTGTATTTCAGTCCACAAGTCCACACTGCAGTGGCAAAGGGTCT

>*Heat shock protein 20* (*HSP20*)

AAATAACACATTTTATTCATGGCGCAAAACTTTCTTAACAAATAAAATACAACATTACTTCATCGCCTTTTGAACGCAACTTGTTAGTGCCCTAAATTATGCTGTAACTAGCTGACATATTCGATGTTTTGCATTCTAAAAGTATTGCTTGAACGACCTAAAGCCGTTTTAAAGCTATATTTCAGTCTTAATTCACTTATTTATACAAATTTATCACTCTTGTTCAACCAGTGCGTAATCTAGACCATTATTGTCATAACCTTTCTCTACTTCGTTGTCTTTCGCTGTTTTGGTTGTGGTTTTTAATTCCTTCTTCCTGATTTTCCCAACATGTGTTGTAGCTTCTACTAAATCCAACTGTTCCAATGGCGTAGCTGATGATGCTTCTAAAGGTGGGGCAGGACTATCCTTTTTCACTACACTTATCTCTTTATCTGTTGTTTGGGAAATCACTTCGGTTTGTTCTGCATTTTCTGGACTTTTCTGTGGTAGTCTTACCTCAATTGGCACTTCTCTTTCCGGCGGTGGTGGAGGTAGCTCATGTTTAGGCACTGATATAGAAAGAACACCATTTTCCTTCAGTACTGCTGTGACTTCCTCTTGCTTGCTTCCAGCTGGCATGATAAATCTCTGCATGAAGTGATTCGCCAAGAACGGTTGATCAATATTCTTATCCTTGTGTTTTCCTTCGACGATAATATAACGGTTCTTCACTTTAATTCTCAATTCATCAGGCTTAAATCTTCTGACGCTTAGGTTCACTTGGAATTTCTCTTTGTCGGACACTATATGTGGTTCTTCAAGTGCGTCTTCGACTTCTAAAGCTGCCGCTGCTCTGATTAATTGACTCCAAGGCCACATATAATGGTAGGCCAATGTGTGGGATAGGTGTTTGTCAATGATGGCAAAAGGATCGTCAACTCTATGCTTCCTGCCTTCATGACAGGTTGCGGTTGCTAGGAATGCGAGTACTAAGATATATTTCTGCATCGTTCGGCTTGCG

>*Ubiquitin-specific protease* (*USP*)

TGTACATTGGTGTAGAAGGTAAAATATAACTGTCATTATAACACTAGGTGGCGGGTTCTAAAAGTGCTGAGTCCCAGCATTGGACACAATAGGTCAGTTCTAGAAGTGTTGAGACCCAGCGTTGGGCAGATGGTCCCGGGTTCGTTCGTAGAACAGTAGGTAGGCAGCACAGCGGAGAACCTCTTGTAGAGAAACTTCATGTACCAGTGTGTCTGATGTGTACCACCATCTTTTGGCTTCAAATCCATTTCCTCTCCGATAAGTAGCGAAATGTCCGGAGCGTGGCCCTCCCACGTGCACCACCACCGCCGTCAGTCTGTAGAGGGCGCCTTCCCCGGCGGCGGGCTCCGCGGTGTTCAGCGTCGATAGCCCCATGCGGAGACGACCCTCGCCCACCAGGGACACCAGGTCTAACTTAGGCTGCTGGACTTGTGCGTAGGGCGCCATGGAGAGCGTCTCTGGGAACGAGACGAACTCTCCGCGCTTGTTGAGGGCACCGCCGCGCCATTCCACTCGCCCGATGTGTAGGCACAGGCACACTGGTAACTTGCCAAAGCTAACAGTTCTTATATGCTTAGTATAGGCGGTGGGGGAGGCTGAAGGTGCTGGATCTGCCGTTTCTGGACAGCATTTTGTACAGCGGACACCTTTCACCATCTCCGGCGCTGTGAAGGCTCGCAGGAGACCAGCAAGACTGAAGGAGCCAGTGAGCCCCGTGCTGGCGTTGGTCATCGAAAGTGAAATACTATCAAACTTATCGTACCGGATCGGACTCTTGTGTGAGCACACAGTGCATTGAAGACGCGAGGCCAAGGTTCCTCTGAAGGGAGGCGCCGGAGGTGGTCTGGCAGGGGCCGGCGTCACCCAGCGCCGCCCCACAGAGTTCAGGTGGCACAGGGATCGAGAGACACCCTTCGTTAGACGGGGCATCGTTACCTCTGGTTCCTCCAAATCTGGTTCATCAGTAGGCGCAGCTGAGGCGGGTCTCAGAGACAGCGCGGAAGGTGTCGGCGGGGTGGAATCAGCTCTCAAGGGGTGAGGCACGGCGGGAGGGGACGCGGGTGATGCTAGGGCAGACCATGCGCGATCCCCGCCCAGACCTAGCGCGTCTGAGAGGCAGCCCGGCTTCCTAGCCATAGCCGTGGTCTCCTCTTCAATACAAGAGAGCAGCACGTGGAGCAGTTCGTGAGCGTCCTGCTGGTCCGCCGGCACCACCCAGCCGGCAGCGCGCAGAGACTGTAGCACTCCTAGTGGACATACTGGAGTGCCCCGGGCTGACTCGTGGGTGCCGTTTACAACTTCAATCACTGTGTATAGCGTAGTTATCATACTGTTGTGACTATCAGCTTTCGCGTACTTCTTGAGCCATTCAATAAAAGTGGGACAGGCAGCCAGCGCCTGCAGAAGTGTGTTTAGGAAACATGTCCTGCCCAAGTTTTGAAGTCCTGCGATTTGTCCACGACGTTTCCGCACTTTTGGAGCTCCACCAGGACCCCAAAGCACAAAAGCACCCACCACTACTGCAGCAGTCAAGCCCGCCGCCACTAAAATTCTGTCTCCACCATCCATAGTCCTGGTAGGATGAATAAACCTAACACTATTTTTTACATATTAACGTCCACACATCGTTAAAACTAACTAATAATTCATTAAAATATTAATAAGCTGTTATTGCGTTTTTCCAAATTAAATTAACCATGGAAAAGAAAATCAGTCAGATCCGCGGGTTGACA
